# Tuning tRNAs for improved translation

**DOI:** 10.3389/fgene.2024.1436860

**Published:** 2024-06-25

**Authors:** Joshua L. Weiss, J. C. Decker, Ariadna Bolano, Natalie Krahn

**Affiliations:** Department of Biochemistry and Molecular Biology, University of Georgia, Athens, GA, United States

**Keywords:** tRNA engineering, synthetic biology, translation, genetic code expansion, noncanonical amino acid, directed evolution, rational design, tRNA therapeutics

## Abstract

Transfer RNAs have been extensively explored as the molecules that translate the genetic code into proteins. At this interface of genetics and biochemistry, tRNAs direct the efficiency of every major step of translation by interacting with a multitude of binding partners. However, due to the variability of tRNA sequences and the abundance of diverse post-transcriptional modifications, a guidebook linking tRNA sequences to specific translational outcomes has yet to be elucidated. Here, we review substantial efforts that have collectively uncovered tRNA engineering principles that can be used as a guide for the tuning of translation fidelity. These principles have allowed for the development of basic research, expansion of the genetic code with non-canonical amino acids, and tRNA therapeutics.

## 1 Introduction

tRNAs play an essential role in translation, mediating the conversion of nucleic acid templates into protein products. They facilitate this by binding a programmed amino acid substrate before transferring it to the ribosome for incorporation into a peptide. During this process, tRNAs make specific interactions with several binding partners: elongation factors, mRNA, and multiple sites within the ribosome. As such, tRNAs directly influence translation and are a prime scaffold for its engineering. This engineering primarily addresses three fundamental applications: i) basic research, ii) genetic code expansion (GCE) in synthetic biology, and iii) tRNA therapeutics.

Basic research on understanding protein synthesis has identified a plethora of information regarding tRNA sequence and structure. The specific elements in the tRNA sequence that promote or deter interaction with a binding partner are termed identity or anti-determinant elements, respectively. The interactions between tRNAs and their cognate aminoacyl-tRNA synthetase (aaRS) in particular have been extensively studied (Giegé and Eriani, 2023) and are an important first step in translation. Still, aminoacyl-tRNAs (aa-tRNAs) must be efficiently incorporated into the ribosome to complete translation. Natural translation machinery is flexible enough to efficiently incorporate the natural amino acids which contain a broad range of chemical properties. However, ribosomal incorporation of non-canonical amino acids (ncAAs), important for widespread applications in synthetic biology, drug discovery, and basic research, are inherently poor ribosomal substrates that can dramatically lower ribosomal translation efficiency. Chemical synthesis via non-ribosomal peptide synthetases (Walsh et al., 2013; Süssmuth and Mainz, 2017) and post-translational modification enzymes (McIntosh et al., 2009) are some alternate strategies to bypass ribosomal synthesis of these amino acid derivatives. However, these strategies are not compatible with screening vast libraries for structurally unique or medically relevant peptides, which is a possibility in a ribosome-based (Yamagishi et al., 2011; Goto and Suga, 2021; Katoh and Suga, 2022a). Through tRNA engineering, hundreds of traditionally ribosome-incompatible substrates have been permitted for ribosomal installation with varying levels of success (Katoh and Suga, 2022a; Sigal et al., 2024). Additionally, nearly 10% and 50% of pathogenic genetic conditions result from aberrant stop codons (Mort et al., 2008) and missense mutations (Katsonis et al., 2014; Stenson et al., 2017), respectively. This has led to substantial research aimed towards developing modified tRNAs for efficient, orthogonal, and safe readthrough of improper gene sequences (Dolgin, 2022; Anastassiadis and Köhrer, 2023; Coller and Ignatova, 2024).

The process of tRNA engineering itself can be broadly divided into engineering for i) improving the orthogonality of an aaRS:tRNA pair, and ii) moderating the aa-tRNA’s journey to and through the ribosome. Briefly, the former objective optimizes aaRS:tRNA interactions to ensure orthogonality and production of the desired aa-tRNAs (Soye et al., 2015; Tamaki et al., 2018; Melnikov and Söll, 2019; Krahn et al., 2020b; Ganesh and Maerkl, 2022; Kim et al., 2024). The latter objective optimizes aa-tRNA interactions with elongation factors, mRNA, and the ribosome to mediate initiation (Tharp et al., 2021b; Katoh and Suga, 2023a; Katoh and Suga, 2023c) or elongation fidelity (Uhlenbeck and Schrader, 2018; Sigal et al., 2024). Our review addresses tRNA engineering principles for the latter processes as a roadmap for modulating tRNA incorporation into the ribosome. We will discuss four major roadblocks involved in this endeavor: i) initial acceptance of an aa-tRNA into the ribosome (P-site for initiation, A-site for elongation), ii) proper codon-anticodon interaction, iii) peptide bond formation and peptidyl transfer efficiency, and iv) translocation of the tRNA. Through this discussion we will highlight the tRNA elements found to overcome these challenges.

### 1.1 tRNA structure

**FIGURE 1:**
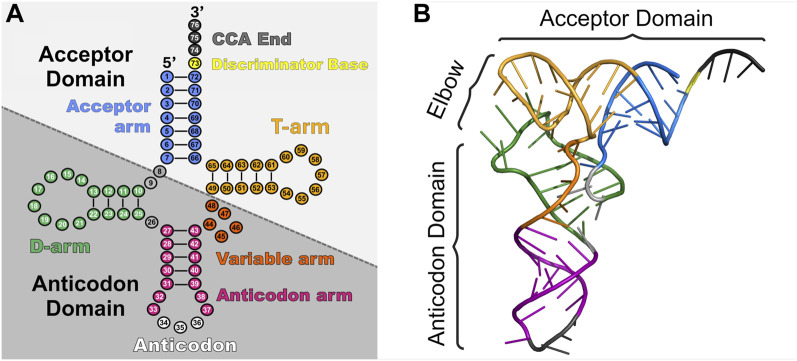
Schematic of tRNA structure. tRNAs consist of two major domains (acceptor domain and anticodon domain) and five stem-loop arms (acceptor arm (blue), D-arm (green), anticodon arm (pink and white), variable arm (orange), and T-arm (gold)) which play different roles in translation. **(A)** Cloverleaf 2D structure of tRNA^Asp^ (PDB: 6UGG) highlighting the major domains and regions. **(B)** 3D stick representation of tRNA^Asp^ with domains highlighted.

A tRNA’s overall three-dimensional “L-shape” and specific nucleotide sequence dictates its interactions with binding partners and overall function. Canonical tRNAs vary between 76 and 100 nucleotides (Krahn et al., 2020a) and have a highly conserved “cloverleaf” secondary structure (Holley et al., 1965; Bolton and Kearns, 1975; Berg and Brandl, 2021) divided into five stem-loop arms based on internal base pairing: the acceptor stem, D-arm, anticodon arm, variable arm, and T-arm (Figure 1) (Giegé et al., 2012). The acceptor stem contains the 3′ conserved CCA sequence for attachment of the amino acid and discriminator base that participates in conferring aaRS specificity. The D-arm is named after a conserved, modified **d** ihydrouridine base that aids in stabilizing the tertiary tRNA structure (Maglott et al., 1999). The anticodon arm contains a three-base anticodon sequence for decoding the mRNA surrounded by other nucleotides that impact its efficiency and binding to aaRSs. The T-arm is named after its universally conserved **t** hymine-pseudouridine-cytidine sequence (TΨC) that aids in ribosome and elongation factor interactions (Holley et al., 1965; Nissen et al., 1996). Finally, the variable arm varies in length and function, participating in binding of certain translation factors and/or impacting overall tRNA flexibility during mRNA translation (Sun and Caetano-Anollés, 2009; Krahn et al., 2020a; Prabhakar et al., 2022).

Structure conservation in tRNAs is also found in 3D (Figure 1), as they twist and fold into an L-shape (Kim et al., 1974; Giegé et al., 2012). This occurs by coaxial stacking of the acceptor stem and T-stem with the D-stem and anticodon stem. Furthermore, the variable loop and D-stem undergo intramolecular tertiary interactions with the D-loop and T-loop (Biela et al., 2023). This newfound L-shape consists of the acceptor domain (acceptor arm and T-arm), elbow region (D-loop and T-loop) (Zhang and Ferré-D’Amaré, 2016), and anticodon domain (D-stem, variable loop, and anticodon arm). The acceptor domain is primarily responsible for binding to its specific aaRS followed by the elongation factor (EF-Tu in prokaryotes, eEF1A in eukaryotes) for transport to the ribosome. The elbow region notably interacts with all three of the ribosome’s tRNA binding domains and an extensive list of RNA and proteins that help mature and modify tRNAs (Zhang and Ferré-D’Amaré, 2016). Decoding of the mRNA occurs through the anticodon domain (Yarus et al., 1986).

In addition to conserved structural features, tRNAs are also heavily modified to further define their function, including all three stages of translation (Agris, 2008; Grosjean et al., 2010; Phizicky and Alfonzo, 2010; Phizicky and Hopper, 2010; Wang and Lin, 2023). In fact, tRNAs are the most extensively modified cellular RNA with about 12% of nucleotides modified (an average of eight modifications per bacterial tRNA and 13 per eukaryotic (Zhang et al., 2022) and around 100 distinct modifications identified in total (Machnicka et al., 2014; Cappannini et al., 2024). Some bases like the 3′ CCA end and discriminator base are never modified, while others are modification hotspots, such as nucleotides in the elbow region (Yared et al., 2024) and anticodon loop (Lorenz et al., 2017).

Our understanding of natural tRNA structure and its impact on translation is imperative to the design of an effective synthetic tRNA. This information lays the foundation for engineering strategies that have been implemented for this purpose.

### 1.2 Translation process

**FIGURE 2:**
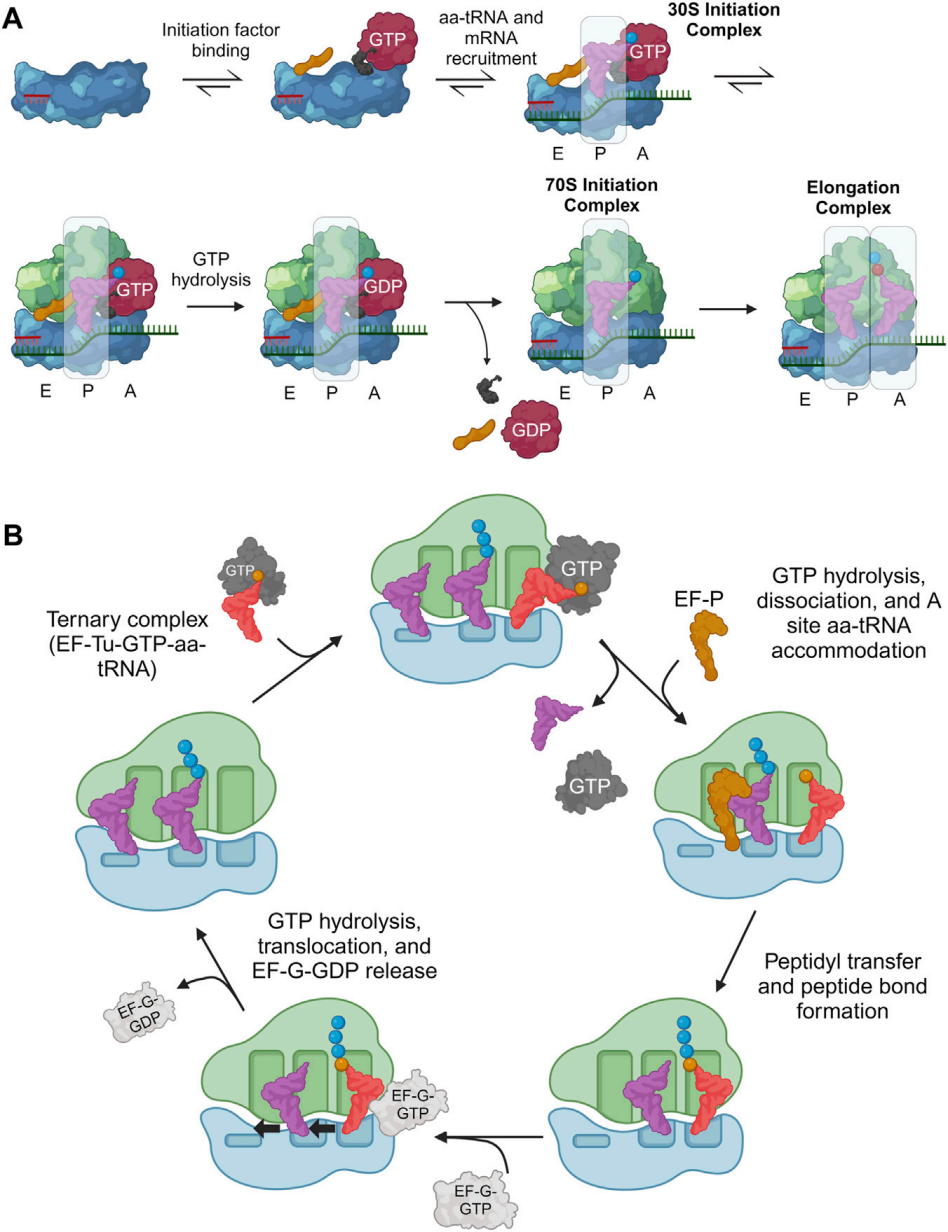
Overview of translation initiation and elongation highlighting important tRNA interactions. During translation, tRNA frequently interacts with both the ribosome and a host of translation factors, playing a central role in all major translational processes. **(A)** Initiation factors (gold, black, and red) help guide the initiator aa-tRNA into the ribosomal P-site to form the 30S initiation complex. Then, the initiation factors release and allow another aa-tRNA to accommodate into the A-site which catalyzes the first peptide bond to form, marking the beginning of elongation. **(B)** In translation elongation, the aa-tRNA must bind EF-Tu/eEF1A to be transported to the ribosomal A/T site. Codon-anticodon interactions between the aa-tRNA, mRNA, and ribosome ensure the correct aa-tRNA is selected before peptidyl transfer between the P-site and A-site tRNAs occur. Additionally, the P-site tRNA binds EF-P/eIF5A when tRNAs are charged with difficult ribosomal substrates to improve peptidyl transfer efficiency. Finally, EF-G/eEF2 interacts with the ribosome and A-site aa-tRNA to catalyze translocation. Created with BioRender.com.

Our focus in translation is tRNA-based, guided in part by structural biology (Chua et al., 2022) which has elucidated many prokaryotic (Rodnina, 2018; Korostelev, 2022; Xu et al., 2022) and eukaryotic translational processes (Neelagandan et al., 2020; Blanchet and Ranjan, 2022; Zhang et al., 2023; Brito Querido et al., 2024). Studies have preferentially investigated the former, resulting in several tRNA engineering strategies developed for prokaryotes. We will focus on the processes of initiation (Figure 2A) and elongation (Figure 2B) as they are most relevant to tRNA engineering efforts.

Initiation of protein synthesis involves anchoring the ribosome onto an mRNA start codon (often AUG) and inserting the first aa-tRNA into the P-site. In bacteria, initiation is composed of two major processes (Milón and Rodnina, 2012; Roy et al., 2017): i) assembly of the 30S initiation complex (30SIC) which involves the binding of initiation factors (IF1, IF2, and IF3), initiator aa-tRNA, and the 30S subunit onto the start codon through interactions with the 30S Shine-Dalgarno (SD) RNA sequence, and ii) release of the initiation factors through the 50S subunit binding to the 30SIC. i) Initiation factors regulate translation initiation, requiring the initiator aa-tRNA, *N*-formylmethionine (fMet)-tRNA^fMet^, to be accommodated into the ribosomal P-site and decode the corresponding AUG start codon (Rodnina, 2018). The GTPase IF2 plays a large role in this selection, through direct contacts with fMet-tRNA^fMet^ localized to the 3′-ACCAAC and the amino acid (Wu and RajBhandary, 1997; Guenneugues et al., 2000; Milon et al., 2010; Roy et al., 2017). ii) After accommodation, the 50S ribosomal subunit binds the 30SIC, catalyzing a series of conformational changes and ultimate release of initiation factors, to form the mature 70S initiator complex (70SIC) (Rodnina, 2018). As with many natural processes, there are exceptions to the general scheme of translation initiation including prokaryotic mRNA that lack a SD sequence, alternate starts codons (Tharp et al., 2020b) or the lack of a 5′UTR (Rodnina, 2018). However, the standard SD-led initiation at an AUG start codon, as described above, is almost exclusively explored with respect to initiator tRNA engineering.

Eukaryotic initiation resembles the path in prokaryotes with eIF1A, eIF2, and eIF1 homologous to bacterial IF1, IF2, and IF3, respectively (Brito Querido et al., 2024). In this process, the 43S pre-initiation complex (PIC) is formed through binding of initiation factors eIF1, eIF3, eIF1A, and eIF5, and a ternary complex [GTP-bound eIF2 complexed to the initiator aa-tRNA (Met-tRNA_i_^Met^)] to the 40S subunit (Lapointe et al., 2022). In eukaryotes, there is no SD sequence, therefore the 43S PIC is recruited to the 5′ end of an mRNA by the mRNA-bound eIF4F complex with the help of eIF4A and eIF4B to form the 48S PIC. The 48S PIC then scans the 5′UTR for a start codon (Wang J. et al., 2022). Upon recognition of this codon, most initiation factors are released, and the 60S subunit binds (catalyzed by eIF5B) to produce the 80SIC with a decoded AUG start codon in the ribosomal P-site (Brito Querido et al., 2024).

After translation initiation, the ribosome complex is ready to grow the nascent polypeptide chain. This is done through a well-conserved cyclic process to incorporate elongator tRNAs and form peptide bonds between amino acids (Dever et al., 2018). In prokaryotic systems, GTP-bound EF-Tu supports aa-tRNAs to the 70S ribosomal A/T-site where it decodes the exposed mRNA codon to promote tRNA accommodation into the ribosomal A-site and EF-Tu release. A peptide bond is then formed between the accommodated A-site and P-site aa-tRNAs (including de-acylation of the P-site tRNA) within the ribosomal peptidyl transferase center (PTC). Elongation factor P (EF-P) binds near the E-site to accommodate this reaction. Afterwards, the ribosome translocates along the mRNA by one codon (moving the tRNA species to the E- and P-sites, respectively), thereby opening the A-site to accept another aa-tRNA and repeat the elongation cycle (Xu et al., 2022). Elongation factor G (EF-G) binds near the A-site to disrupt codon-anticodon interactions and catalyze translocation (Liu et al., 2014; Rodnina et al., 2020). Finally, elongation factor thermostable (EF-Ts) catalyzes GDP-to-GTP nucleotide exchange to recycle the EF-Tu used (Gromadski et al., 2002; Kavaliauskas et al., 2012).

Eukaryotic elongation is similar to that of prokaryotes. GTP-bound eEF1A binds an aa-tRNA and is transported to the eukaryotic 80S ribosome (Dever et al., 2018). Base pairing interactions between the aa-tRNA anticodon and A-site codon catalyzes GTP hydrolysis of eEF1A (Shao et al., 2016), leading to its dissociation from the ribosome and aa-tRNA accommodation (Jobe et al., 2019). Peptide bond formation and translocation is similar to that of prokaryotic systems (Ben-Shem et al., 2011). eIF5a (bacterial EF-P ortholog) also binds the E-site and interacts with the acceptor arm of the peptidyl-tRNA to promote favorable substrate positioning and efficient peptide bond formation (Gutierrez et al., 2013; Melnikov et al., 2016b; Shin et al., 2017). Translocation is enhanced by eEF2, and eEF1B (orthologs of EF-G and EF-Ts, respectively) to catalyze a similar nucleotide exchange as EF-Ts to renew eEF1A for another cycle of elongation (Dever et al., 2018).

## 2 Acceptor domain engineering for improved EF-Tu interactions

### 2.1 Basis of tRNA engineering for improved interactions with EF-Tu

**FIGURE 3:**
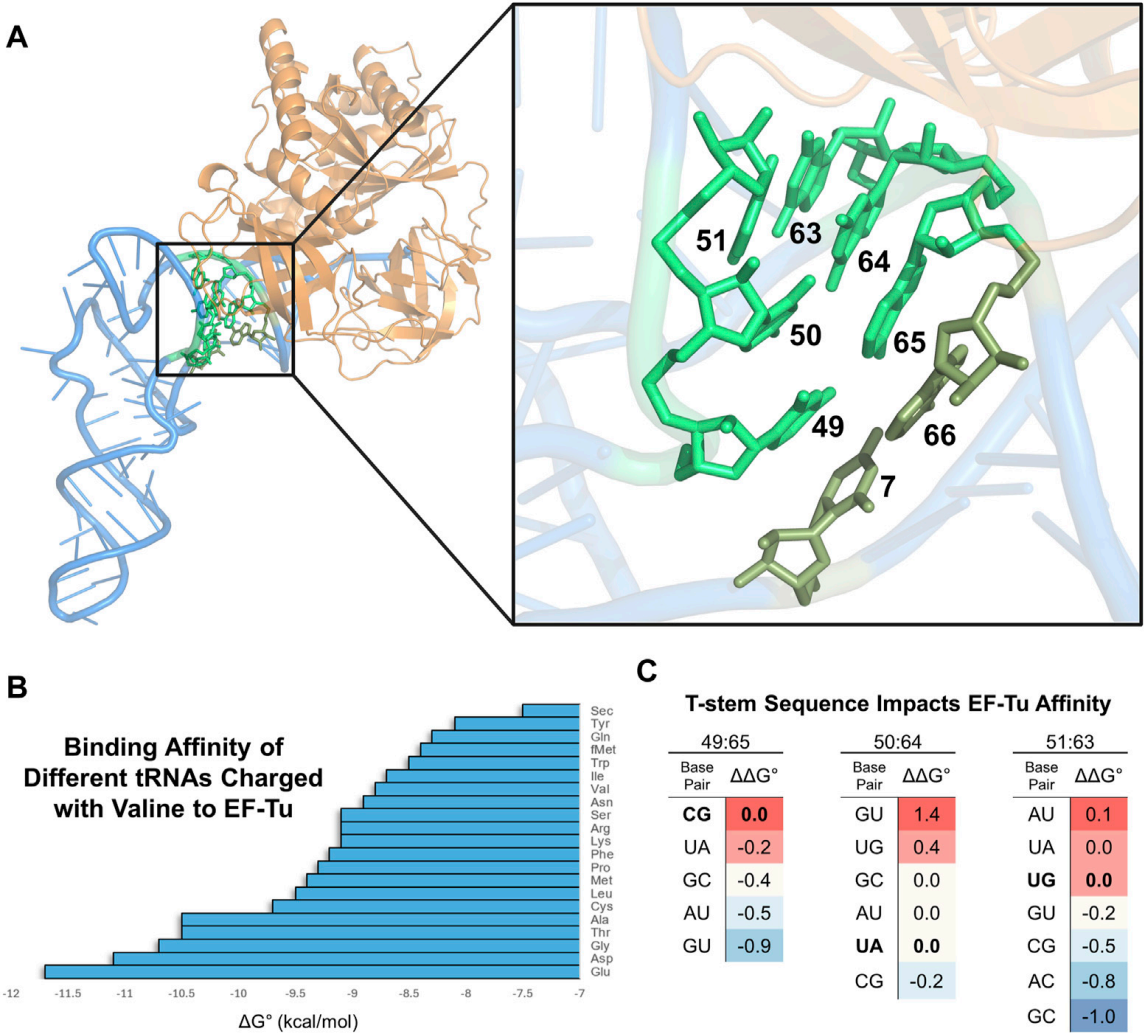
tRNA interacts with EF-Tu to moderate ribosome acceptance of aa-tRNAs. **(A)** Visualization of *E. coli* Cys-tRNA^Cys^ (blue) bound to EF-Tu (orange) (PDB: 1B23). The black box highlights EF-Tu interactions with the acceptor domain of the tRNA. A zoom-in on the box shows the specific bases involved (highlighted in bright green). Base pair 7:66 (olive green) contributes least to the affinity, while the other three pairs contribute more. **(B)** Graph showing the different binding affinities of natural elongator valine-charged *E. coli* tRNAs to EF-Tu (Asahar and Uhlenbeck, 2002: Copyright (2002) National Academy of Science, United.States). **(C)** Different T-stem base pairs [highlighted in panels **(A, B)**] modularly impact aa-tRNA binding to EF-Tu. The bolded base pairs for each of the three T-stem base pairs represent the natural sequence when calculating the relative ΔΔG° to compare binding affinity differences. The intensity of red and blue shading indicates a base pair with relatively higher or lower affinity to EF-Tu at that position, respectively. Data from Schrader and Uhlenbeck, (2011) with *E. coli* tRNA^Phe^ and figure repurposed from Shrader and Uhlenbeck, (2018).

**FIGURE 4:**
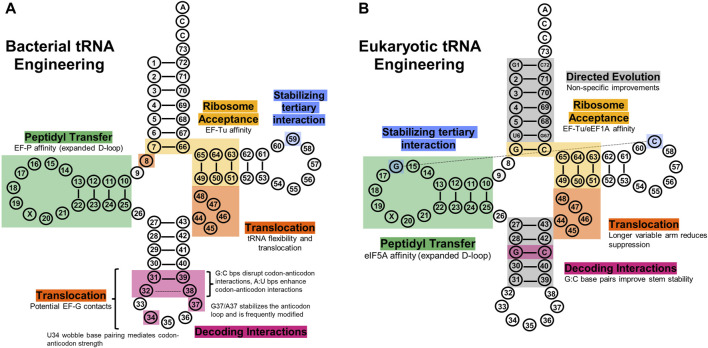
tRNA engineering sites explored to improve translational fidelity. **(A)** Bacterial tRNA regions have shown utility in improving various aspects of translation, such as ribosome acceptance (gold), decoding (pink), peptidyl transfer (green), translocation (orange), and tertiary structure (blue). **(B)** Eukaryotic tRNA regions engineered to improve their translational fidelity. Directed evolution experiments have elucidated the acceptor stem and anticodon stem as sites that non-specifically improve eukaryotic tRNA translational fidelity (grey), and other regions have been rationally engineered similarly to bacterial systems.

Natural aa-tRNAs bind EF-Tu within a remarkably narrow affinity range which is necessary for unbiased transport of all amino acids to the ribosome and smooth mRNA translation (Louie et al., 1984; Ledoux and Uhlenbeck, 2008). This affinity is fine-tuned so that aa-tRNAs bind sufficiently to catalyze translation, but also dissociate at a rate fast enough to permit downstream peptide bond formation (Schrader et al., 2011; Ieong et al., 2014). Extensive research has determined the interface between EF-Tu and aa-tRNA (Figure 3A); importantly, the acceptor domain and amino acid moiety of aa-tRNAs are both responsible for binding EF-Tu (Kjeldgaard et al., 1993; Nissen et al., 1995; Nissen et al., 1996; Nissen et al., 1999; Sanderson and Uhlenbeck, 2007a; Schmeing et al., 2009; Kavaliauskas et al., 2012; Yikilmaz et al., 2014; Fischer et al., 2015; Johansen et al., 2018). Furthermore, both the tRNA sequence and amino acid structure work inversely together to achieve an affinity within this narrow range for optimal translation (Louie and Jurnak, 1985; LaRiviere et al., 2001; Asahara and Uhlenbeck, 2002; Asahara and Uhlenbeck, 2005; Dale et al., 2004; Dale and Uhlenbeck, 2005). Therefore, amino acids with weaker binding affinities are generally attached to tRNAs that have an acceptor domain with high affinity for EF-Tu and vice versa (Figure 3B). A series of mutagenesis-based experiments and computational modeling have further determined that base pairs 49:65, 50:64, and 51:63 within the T-stem determine the tRNA body’s affinity to EF-Tu (Figure 3C), irrespective of the other acceptor domain nucleotides within the interface of EF-Tu (Sanderson and Uhlenbeck, 2007b; Eargle et al., 2008; Schrader et al., 2009; Schrader and Uhlenbeck, 2011). According to this research, these three T-stem base pairs contribute to EF-Tu affinity independently of each other, suggesting that one can accurately predict a bacterial tRNA’s affinity for EF-Tu by summing the known contributions of the individual base pairs. Thus, engineering tRNA T-stems for increased EF-Tu affinity is a common, modular strategy to compensate for the incorporation of ncAAs that can be difficult ribosomal substrates (Figure 4).

### 2.2 Examples of improving EF-Tu binding

#### 2.2.1 Canonical elongator tRNAs

As mentioned, canonical tRNAs have evolved to exhibit a wide range of affinities toward EF-Tu. Therefore, when engineering a tRNA for improved translational fidelity with a ncAA, it can be sufficient to use a natural tRNA with a high affinity for EF-Tu (e.g., tRNA^Glu^). This strategy has notably allowed for the implementation of d-amino acids (AAs), which are pharmacologically relevant, (Feng and Xu, 2016; Melchionna et al., 2016; Wang L. et al., 2022), but notoriously difficult ribosomal substrates (Blackmond, 2010; Fujino et al., 2013; Englander et al., 2015; Fleisher et al., 2018; Liljeruhm et al., 2019; Melnikov et al., 2019). In one case, charging tRNA^Gly^ (fourth strongest affinity to EF-Tu) with various d-AAs led to increased—but variable—single, double, and even triple consecutive insertions (Achenbach et al., 2015). A similar approach utilized a tRNA^Glu^ (strongest natural EF-Tu affinity) variant (Terasaka et al., 2014) to efficiently incorporate up to 10 consecutive d-Ala or d-Ser amino acids into a peptide, and four or five consecutive d-AAs into a macrocyclic peptide *in vitro,* particularly useful for drug discovery (Katoh et al., 2017b; Goto and Suga, 2021). Although tRNA engineering alone demonstrated improved installation efficiency, optimization of IF2, EF-Tu, and EF-G concentrations further improved results—demonstrating the impact and complexity of other factors in cell-free protein synthesis.

#### 2.2.2 *Methanocaldoccocus jannaschii* tRNA^Tyr^ engineering

The *M. jannaschii* (*Mj*) tyrosyl-tRNA synthetase (TyrRS):tRNA^Tyr^ pair is commonly used for GCE in *Escherichia coli* (Xie and Schultz, 2006). After modifying loop regions of *Mj* tRNA^Tyr^ to be orthogonal in *E. coli* (Wang and Schultz, 2001), further research sought to improve its elongation efficiency. Specific nucleotides in the acceptor domain (2:71, 3:70, 6:67, and 7:66 in the acceptor stem, and the entire T-stem) were selected based on a co-crystal structure of EF-Tu with Cys-tRNA^Cys^ (Nissen et al., 1999) and subsequently randomized (Guo et al., 2009). Several effective suppressor tRNA variants emerged which improved the incorporation of ncAAs. In particular, tRNA^opt^_CUA_ was only modified at base pairs 49:65, 50:64, and 51:63, and found to be capable of installing six ncAAs (Young et al., 2010). Notably, the incorporation efficiency differed depending on the ncAA, which holds true with previous comments about the amino acid contacts affecting affinity with EF-Tu.

A separate investigation used a directed evolution approach to improve the ribosomal efficiency for installation of three 3-halo-tyrosines (Maranhao and Ellington, 2017). These efforts focused on modifying the acceptor and T-stem nucleotides that interact with EF-Tu: nucleotides 3, 6, 68, and 71 in the acceptor stem, and base pairs 49:65, 50:64, and 51:63 in the T-stem. Five evolved tRNAs demonstrated 12 to 20-fold higher suppression efficiency for single incorporation of all 3-halo-Tyr variants. However, in the process of evolution, these tRNAs lost varying degrees of orthogonality in the cell, demonstrating that engineering one aspect of a tRNA can impact other tRNA functions.

#### 2.2.3 tRNA^Pyl^ engineering

Pyrrolysine (Pyl) is the 22nd proteinogenic amino acid found in archaea (Tharp et al., 2017) and some bacteria (Matassi, 2017) with its own incorporation machinery: pyrrolysyl-tRNA synthetase (PylRS) and its cognate tRNA (tRNA^Pyl^). This system has been extensively researched for its use in GCE because of the promiscuity of the PylRS binding pocket and the orthogonality of the archaeal PylRS:tRNA^Pyl^ protein synthesis system in *E. coli* (Tharp et al., 2017) and eukaryotic systems (Chin, 2014; de la Torre and Chin, 2021). Significant research has focused on expanding the substrate capacity of PylRS and engineering PylRS:tRNA^Pyl^ pairs to be mutually orthogonal (Wan et al., 2014; Beattie et al., 2023; Gong et al., 2023). However, others have focused on optimizing tRNA^Pyl^ for improved translational fidelity (Wang J. et al., 2016). In one case, rational engineering of the T-stem and acceptor stem in *Methanosarcinaea barkeri* tRNA^Pyl^ resulted in improved binding to PylRS, and *E. coli* EF-Tu for incorporation of *N*(ε)-acetyl-l-lysine (AcK) (Fan et al., 2015). Small libraries of tRNA mutants were created at base pairs 2:71, 3:70, 6:67, and 7:66 in the acceptor stem, and 49:65 and 50:64 in the T-stem. It was found that changes to base pairs 7:66, 49:65, and 50:64, which are well-known EF-Tu affinity determinants (Asahara and Uhlenbeck, 2002), benefited from mutations. The best tRNA variant, tRNA^Pyl-opt^, with G7:C66, U49:A65, and G50:C64 had a ∼3-fold improvement in AcK incorporation into sfGFP and human histone H3. tRNA^Pyl-opt^ was also used to incorporate two different ncAAs simultaneously at different sites within a protein (Zheng et al., 2018). These results collectively demonstrate the effectiveness of T-stem engineering to incorporate ncAAs. Combined with its orthogonality in heterologous systems, tRNA^Pyl^ is a responsive platform that can be fine-tuned to efficiently accommodate various ncAAs.

#### 2.2.4 tRNA^Sec^ engineering

Selenocysteine (Sec) is the 21st genetically encoded amino acid, found in all three domains of life, and can imbue proteins with unique properties such as overoxidation resistance, diselenide bond formation, and ability to be chemically modified (Arnér, 2010; Reich and Hondal, 2016; Mousa et al., 2017). Unlike the other natural amino acids, the mechanism for incorporating Sec into the ribosome involves additional steps and translation factors, including a specialized elongation factor, which makes site-specific Sec incorporation into proteins a formidable challenge (Yoshizawa and Böck, 2009; Chung and Krahn, 2022; Wright and O’Donoghue, 2024). The extended, 8 bp acceptor stem of tRNA^Sec^ is a distinguishing feature that allows recognition by SelB and was originally hypothesized to prevent it from binding to EF-Tu (Baron and Böck, 1991). It was later shown that EF-Tu is capable of binding extended acceptor stems, albeit at a lower affinity, and rather it is the antideterminant sequence in tRNA^Sec^ that specifically blocks EF-Tu binding (Rudinger et al., 1996). The antideterminant sequence in tRNA^Sec^ consists of base pair C7:G66 in the acceptor stem and base pairs G49:U65 and C50:G64 in the T-stem—the same base pairs that are modified to improve EF-Tu affinity in other tRNAs. Thus, these base pairs act as a double-edged sword for EF-Tu binding; they can drastically improve and inhibit tRNA affinity for EF-Tu (Rudinger et al., 1996; Giegé and Eriani, 2023).

With this information, it suggests that tRNA^Sec^ could be engineered to be recognized by EF-Tu instead of SelB. This would open the possibility for overexpression of selenoproteins and site-specific installation of Sec to harness its enhanced chemical properties. To achieve this, hybrid tRNAs were created between tRNA^Sec^ and tRNA^Ser^ (tRNA^UTu^, tRNA^SecUx^, tRNA^UTuX^, and tRNA^UTuT^ variants) by several groups. These hybrid tRNAs successfully demonstrated the possibility of EF-Tu-mediated Sec insertion (Aldag et al., 2013; Miller et al., 2015; Thyer et al., 2015; Fan et al., 2018).

The earliest example (tRNA^UTu^, Aldag et al., 2013) utilized tRNA^Ser^ as the major scaffold; it retained the recognition elements for EF-Tu (G7:C66, C49:G65, and A50:U64), and incorporated the rest of the extended acceptor stem of tRNA^Sec^ necessary for SelA recognition (to convert Ser-tRNA^Sec^ to Sec-tRNA^Sec^). This created a 13 bp acceptor domain conducive for SelA recognition, but with EF-Tu identity elements already present in tRNA^Ser^ to mimic the translation pathway of canonical AAs. Although a great leap forward in simplifying Sec’s translation pathway, this tRNA resulted in low selenoprotein yield and significant serine misincorporation (as EF-Tu still recognizes Ser-tRNA^UTu^) (Aldag et al., 2013). Optimizing Ser to Sec conversion became the focus of subsequent rational tRNA engineering efforts that were outside of the acceptor domain (tRNA^UTuX^, Miller et al., 2015).

Another group utilized *E. coli* tRNA^Sec^ as a scaffold to design a tRNA with heightened EF-Tu binding affinity (Thyer et al., 2015). They accomplished this through tuning the antideterminant sequence (base pairs 7:66, 49:65, and 50:64) known to govern EF-Tu affinity (Rudinger et al., 1996; Schrader et al., 2009; Schrader and Uhlenbeck, 2011). Additionally, the authors hypothesized that tRNA^UTu^ demonstrated Ser misincorporation due to reduced SelA contacts with the D- and T-loops of tRNA^Sec^ as shown in the crystallized SelA structure (Itoh et al., 2013a; Thyer et al., 2015). Results indicated that G7:C66, U49:G65, and C50:U64 modifications on the tRNA^Sec^ scaffold (tRNA^SecUx^) greatly improved EF-Tu affinity and subsequent Sec insertion efficiency compared to tRNA^UTu^.

Later, another group re-engineered tRNA^UTu^ by creating a small library of variants combining different tRNA^Ser^ and tRNA^Sec^ sequence segments. The segments tested were the acceptor stem, T-stem, and nucleotide 59 in the T-loop from either tRNA^Ser^ or tRNA^Sec^. Nucleotid as tested because it is a conserved base (Romby et al., 1987; Lowe and Chan, 2016) relevant for maintaining 3D L-shape (Sanbonmatsu et al., 2005; Pan et al., 2008; Itoh et al., 2013b) and SelA binding (Fischer et al., 2016). This strategy resulted in many tRNA variants with modified T-stems, increasing their affinity to EF-Tu (Lajoie et al., 2013). However, the best performing tRNA (tRNA^UTuT6^) was formed by only changing A59C from the original tRNA^UTu^ (Fan et al., 2018). This suggests that other T-arm nucleotides can contribute to translational fidelity, perhaps by improving interactions with SerRS, SelA, EF-Tu, and/or improved tertiary tRNA structure. The unique batch of tRNA variants from this study provided an unclear pattern on how to increase EF-Tu affinity.

Combined, these efforts demonstrate that Sec translation can be rewired for elongation by EF-Tu through focused engineering on the tRNA acceptor domain. However, the efficiency of these changes is limiting, most likely due to the fact that tRNA^Ser^ and tRNA^Sec^ have evolved for specific interactions with their translation machinery. Therefore, as discussed below, there are alternative strategies that can be taken to increase translation efficiency.

### 2.3 The curious case of EF-Ts

EF-Ts canonically function by binding EF-Tu-GDP and catalyzing GDP dissociation, allowing EF-Tu to rebind GTP and deliver another aa-tRNA to the ribosome (Miller and Weissbach, 1970; Weissbach et al., 1970; Miyajima and Kaziro, 1978; Pedersen et al., 1978; Romero et al., 1985; Wang et al., 1997). However, there is also research indicating that EF-Ts may have a more direct role in elongation by interacting directly with the aa-tRNA (Romero et al., 1985; Schwartzbach and Spremulli, 1991; Bubunenko et al., 1992) by i) regulating ternary complex formation and dissociation in response to cellular growth or stress signals, and ii) forming quaternary complexes that dramatically increase the rate of protein synthesis (Burnett et al., 2013; Burnett et al., 2014). Although there is no solved structure for aa-tRNA-EF-Tu-GTP-EF-Ts, it has been suggested that direct interactions between EF-Ts and the ternary complex are possible (Kawashima et al., 1996; Gromadski et al., 2002; Burnett et al., 2013) and may bind the aa-tRNA acceptor stem and elbow region in a conformation similar to the previously characterized *E. coli* EF-Tu-EF-Ts complex (Kawashima et al., 1996). It is unclear exactly how EF-Ts may interact with the complexed aa-tRNA, and whether tRNA engineering to improve favorable quaternary interactions with EF-Ts is a viable strategy for improving translational fidelity.

## 3 Improving codon-anticodon interactions

After the ternary complex has delivered aa-tRNA to the ribosome, the complex undergoes a series of interactions to ensure proper acceptance into the A-site for peptidyl transfer (Rodnina et al., 2017). Decoding efficiency essentially relies on a careful balance of codon-anticodon interaction strength and the ability of tRNA to adopt a proper conformation, both of which have been evolutionarily tuned, in part by tRNA modifications, to accommodate different codons and aa-tRNA combinations (Lorenz et al., 2017; Uhlenbeck and Schrader, 2018). The principles guiding this idiosyncratic tuning are less complete than the principles that have been shown to guide EF-Tu-related tRNA engineering efforts (Figure 4). Regardless, there has still been an exciting amount of information hinting at tRNA engineering principles to help improve their decoding mechanics, focused overwhelmingly on bacterial systems.

### 3.1 Engineering the anticodon arm for improved translational fidelity

While the anticodon sequence itself determines a substantial proportion of the codon-anticodon interactions within the ribosome (Rozov et al., 2016; Loveland et al., 2017; Rodnina, 2023), it is also well-known that surrounding nucleotides and base pairs within the anticodon arm influence these dynamics to uniformly tune the different strengths of codon-anticodon interactions to ensure uniform decoding efficiency and rate of mRNA translation (Olejniczak et al., 2005; Olejniczak and Uhlenbeck, 2006; Ledoux and Uhlenbeck, 2008). This covariance between the anticodon sequence and other anticodon arm elements is known as the “extended anticodon” (Yarus, 1982; Yarus et al., 1986; Raftery and Yarus, 1987; Kleina et al., 1990; Auffinger and Westhof, 2001; Murakami et al., 2009; Schmeing et al., 2011; Shepotinovskaya and Uhlenbeck, 2013; Cervettini et al., 2020). Specifically, it has been shown that the identities of positions 32, 37, 38, and at least two base pairs in the anticodon stem are well-conserved (Saks and Conery, 2007).

The flexibility of the anticodon stem-loop, largely influenced by base pair 31:39 and non-canonical interactions between nucleotide 32:38 (Auffinger and Westhof, 1999), impacts this decoding efficiency and codon recognition specificity (Olejniczak et al., 2005; Ledoux and Uhlenbeck, 2008; Sigal et al., 2024). “Stiffer” anticodon arms result from G:C base pairs at these positions, generally distorting the canonical anticodon loop conformation and disrupting the binding of GC-rich codons (Olejniczak et al., 2005; Olejniczak and Uhlenbeck, 2006; Murakami et al., 2009), whereas weaker interactions, including the rare A32:U38 interaction, allows easier codon-anticodon base pairing (Ledoux et al., 2009; Schmeing et al., 2011; Grosjean and Westhof, 2016). Therefore, tRNA sequences are seemingly tuned for optimal bending in the ribosome to off-set the strengths of different codon-anticodon interactions (Shepotinovskaya and Uhlenbeck, 2013).

A more recent example (Katoh and Suga, 2024) leveraged these principles to further improve the translational efficiency of an engineered tRNA platform (discussed below) to incorporate especially difficult ribosomal substrates that had eluded previous tRNA engineering efforts (Lee et al., 2022). Crucially, screening the anticodon stem of the scaffold tRNA^Pro1E2^ (Katoh et al., 2017a) led to different tRNA^Pro1E2^ variants each uniquely optimized to one of five different anticodon sequences. Then, anticodon loop mutations at positions 32, 33, 37, and 38 were introduced according to the native *E. coli* anticodon sequences leading to further improvement of ncAA incorporation efficiency. Combined, these improvements allowed for multi-site incorporation of βAAs at five different codons *in vitro* and the first reported ribosomal installation of 10 consecutive β-homophenylglycine (βPhg) residues. The G:C or C:G base pair at 31:39 was conserved across all codons, along with a conserved A37.

However, it is worth noting that mutational, structural, and bioinformatic has also identified positions dispersed throughout all arms of bacterial tRNAs that influence proper bending and decoding specificity (Saks and Conery, 2007; Schmeing et al., 2011; Shepotinovskaya and Uhlenbeck, 2013). In this way, the “extended anticodon” truly extends throughout the entire tRNA, potentially complicating tRNA engineering efforts when focusing on multiple tRNA regions.

### 3.2 Anticodon arm modifications influence decoding

tRNAs are frequently post-transcriptionally imbued with diverse modifications that drastically impact their structure and function (Lorenz et al., 2017; Suzuki, 2021; Cappannini et al., 2024; Yared et al., 2024). tRNAs exhibit diverse modifications in all arms, but they are most frequent at the tRNA core and anticodon arm (Cantara et al., 2011; Smith et al., 2024). Anticodon arm modifications in particular have thus been targets for uncovering tRNA translation fidelity principles as an “unrealized genetic code” to complement the tRNA sequence itself (Agris et al., 2017). In particular, modifications at positions 32, 34, and 37 are frequent and chemically varied, especially at the first two positions (Jühling et al., 2009).

The most well-known examples of post-transcriptional modifications impacting decoding are those that occur at position 34, the “wobble” base (Crick, 1966; Agris, 1991; Demeshkina et al., 2012). In some cases, the modification status at position 34 expands wobble pairing to all four mRNA nucleotides at the third codon position (Rogalski et al., 2008; Bloom-Ackermann et al., 2014). Other modifications have been shown to impact decoding specificity or even completely disable codon binding altogether (Kurata et al., 2008; Kumbhar et al., 2013; Nedialkova and Leidel, 2015; Nakano et al., 2016; Song et al., 2022). A34 is also almost universally modified to inosine to expand decoding potential to C, U, and A (Crick, 1966). Position 37 does not directly interact with the codon, but is often a purine frequently modified to stabilize Watson-Crick base pairing at the first two positions of a codon (Väre et al., 2017). Moreover, position 32 modifications generally help to create non-canonical interactions at base pair 32:38 to stabilize the U-turn structural motif of the anticodon arm relevant for ribosomal A-site acceptance and codon discrimination (Auffinger and Westhof, 1999; Cantara et al., 2012; Väre et al., 2017). In addition to the anticodon loop, tRNAs are also often simultaneously modified at anticodon arm positions. These modifications are connected with the anticodon sequence, synergistically functioning to alter tRNA structure and decoding efficiency (Nishimuka, 1972; Jühling et al., 2009; Cantara et al., 2012; Grosjean and Westhof, 2016; Väre et al., 2017). However, the exact impact of adding or removing a modification across different tRNAs is not well understood, and some unmodified tRNAs can still function (Phizicky and Alfonzo, 2010; Lorenz et al., 2017). One heuristic approximation is that GC-rich decoding tRNAs are modified to reduce binding affinity whereas AU-rich decoding tRNAs are modified to improve codon binding (Uhlenbeck and Schrader, 2018; Sigal et al., 2024). One recent example improved the UAG decoding efficiency of a previously engineered ^opt^tRNA_CUA_^Tyr^ (Young et al., 2010) by replacing adenine with 2, 6-diaminopurine (D) at positions 31 and 36 (Mala and Saraogi, 2022). D is similar to adenine but has an added C_2_ amino group, enabling the formation of an additional hydrogen bond in the D-U base pair compared to A-U pairing, which strengthens codon-anticodon interactions and stabilizes RNA duplexes (Strobel et al., 1994).

It has also been hypothesized that the overreliance on synthetic tRNAs that lack post-transcriptional modifications limits tRNA activity (McFeely et al., 2022); it was recently shown that wild-type (i.e., fully modified) tRNAs significantly outcompeted their synthetic counterparts (3 to 4-fold improvement) for the same cognate sense codons (McFeely et al., 2022). Interestingly, heterologous tRNAs such as tRNA^Pyl-opt^ can also be engineered to reduce dependence on post-transcriptional modifications and improve suppression efficiency (Baldridge et al., 2018).

Combined, tRNA modification engineering will almost certainly aid in producing more effective synthetic tRNAs, although more research is needed to better define fundamental principles.

### 3.3 Engineered quadruplet codons efficiently expand the genetic code

**FIGURE 5:**
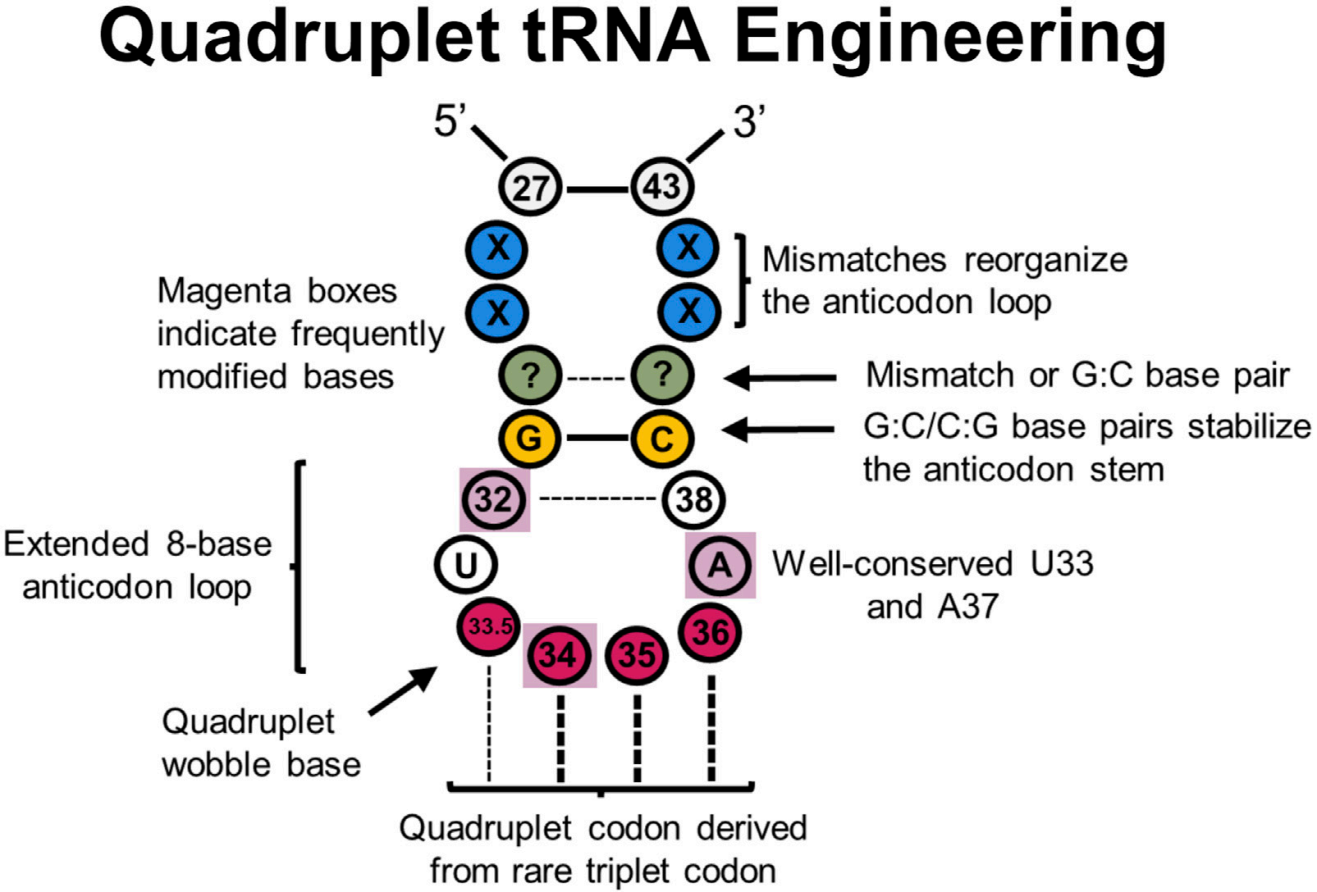
tRNA sites explored in quadruplet tRNAs to improve translational fidelity. Efficient quadruplet tRNAs have a well-conserved G:C/C:G base pair adjacent to the anticodon loop (yellow), A37 and U33 residues, and base pair mismatches near the beginning of the anticodon stem (blue) which adjusts the conformation of tRNAs. Frequently post-transcriptionally modified residues (purple boxes) also impact translational fidelity, although position 33.5 base pairing with a quadruplet codon is not necessary.

Significant research efforts have focused on engineering triplet codons for improved translational fidelity. However, there are limitations for genetic code expansion because triplet codons result in only 64 possible codon combinations, which traditionally encode for 20 proteinogenic AAs (plus Sec and Pyl) and three stop codons. Therefore, there has been significant research efforts toward engineering quadruplet codon-decoding tRNAs—tRNAs that decode four base codons on the mRNA—to vastly expand the available number of empty codons (up to 256 free codons) (Figure 5). Efficient quadruplet codons are also inherently orthogonal to the 64 triplet codons, thereby not requiring codon reassignment.

Such quadruplet decoding, or “programmed frameshifts,” occur in nature, often with an extended 8-base anticodon loop as opposed to the canonical 7-base loop (Riyasaty and Atkins, 1968; Riddle and Roth, 1970; Yourno and Kohno, 1972; Riddle and Carbon, 1973; Bossi and Smith, 1984; Farabaugh, 1996; Moore et al., 2000a; Walker and Fredrick, 2006; Atkins and Björk, 2009; Mohanta et al., 2023). Many experiments have since demonstrated that inserting a single nucleotide into the anticodon loop of a tRNA to create an 8-base anticodon results in moderate quadruplet decoding *in vitro* and *in vivo,* especially in combination with engineered synthetases (Curran and Yarus, 1987; Ma et al., 1993; Hohsaka et al., 1996; Hohsaka et al., 1999; Hohsaka et al., 2001; Moore et al., 2000b; Magliery et al., 2001; O’Connor, 2002; Ohtsuki et al., 2005; Taki et al., 2006; Wan et al., 2010; Chatterjee et al., 2012; Hankore et al., 2019; Dunkelmann et al., 2020; Dunkelmann et al., 2021; DeBenedictis et al., 2022). In a recent report, it was determined that nearly a third of the 20 bacterial tRNA isoacceptors can demonstrate modest quadruplet decoding activity after carrying the insertion of a single nucleotide insertion in the anticodon loop in *E. coli* (DeBenedictis et al., 2022). Additionally, while experiments with quadruplet *M. barkeri* tRNA^Pyl^ using a 7-base anticodon loop were unsuccessful, expanding them to 8-bases provided an avenue for quadruplet decoding (Wang N. et al., 2016). One potential issue with anticodon arm engineering is that the anticodon loop can be an essential identity element for binding respective aaRSs (Melnikov and Söll, 2019). Therefore, engineering the anticodon arm for improved decoding mechanics can also alter aminoacylation specificity and efficiency. Many engineered quadruplet tRNAs thus originate from tRNA^Pyl^ scaffolds as PylRS is known not to interact with the anticodon loop (Nozawa et al., 2009).

Designing quadruplet anticodon sequences derived from rarely-used triplet codons can help with endogenous machinery competing to perform triplet decoding as a general quadruplet tRNA engineering principle (Guo and Niu, 2022). In one case, a GGGU quadruplet tRNA was outcompeted by inserting Gly at the CCC codon in *Xenopus* oocytes (Shafer et al., 2004). In contrast, deriving quadruplet tRNAs from rarely used three base codon sequences in *E. coli* found more success in quadruplet decoding efficiency (Hohsaka et al., 2001). Interestingly, an *in vivo* library of *E. coli* tRNA^Ser^ with randomized 8- or 9-base anticodon loops containing various quadruplet codons (NNNN) found that the most efficient tRNAs decoded CCCU, AGGA, UAGA, and CUAG codons—all of which are derived from rare triplet codons (Magliery et al., 2001). This strategy has also served to successfully incorporate ncAAs in *E. coli* (Anderson et al., 2004; Chatterjee et al., 2014).

As the anticodon arm (and entire tRNA, to an extent) often coevolves with its anticodon sequence, further modification of the anticodon arm outside of the quadruplet anticodon has been shown to improve decoding efficiency. In *E. coli* and mammalian cells alike, *in vitro* evolution of the entire tRNA anticodon arm generated mutants with efficient incorporation of ncAAs at the AGGA codon (Niu et al., 2013). Importantly, sequence convergence of base pairs G28:C36 and G27:C37, along with some base pair mismatch at 26:38 was observed. Combined, this sequence was thought to restructure the anticodon arm to help with A-site binding and translocation. Another investigation (Wang et al., 2014) evolved a series of *M. barkeri* tRNA^Pyl^ mutants for efficient quadruplet decoding with ncAAs using an engineered ribosome (ribo-Q1) (Neumann et al., 2010). tRNAs were randomized at positions 29–33 and 37–41 for four quadruplet codons (AGGA, AGTA, TAGA, and CTAG). The most efficient mutants all shared a G:C or C:G base pair adjacent to the anticodon loop, even though the size of the anticodon loop varied. Position A37 was also conserved among all efficient mutants, which is associated with reading frame maintenance (Agris, 2004). Follow up research varied the same regions in UAGN-decoding *M. mazei* tRNA^Pyl^ to improve quadruplet decoding efficiency and uncover a better mechanistic understanding of quadruplet decoding in *E. coli* (Wang N. et al., 2016). This led to significant quadruplet decoding efficiency improvements and incorporation of ncAAs with two PylRS variants, though quadruplet codon orthogonality was not obtained. Most strikingly, all observed tRNA mutants contained U33.5 regardless of the UAGN codon they decode, validating the previously hypothesized idea that strict Watson-Crick base pairing at all four positions is not required for quadruplet decoding (Guo and Niu, 2022). It is possible that U33.5 impairs interactions at position 32:38 to expand the anticodon loop and permit quadruplet decoding (Maehigashi et al., 2014; Wang N. et al., 2016) and/or preserves an essential U-turn structure in the anticodon loop necessary for translocation (Phelps et al., 2002; Phelps and Joseph, 2005). Importantly, all mutants contained A31G and U39C mutations, resulting in a new G:C base pair which strengthens the anticodon stem, but most mutants did not have Watson-Crick base pairing at pairs 29:41 or 30:40 which may restructure the anticodon arm for more efficient quadruplet decoding. It was also noted that position 33 and 37 were never mutated. While not specifically evolved for mammalian cells, these quadruplet tRNAs were shown to be effective in incorporating a ncAA in HEK 293T cells, suggesting that anticodon arm engineering strategies are applicable to a wide range of systems (Chen et al., 2018). Additional research (Mills et al., 2021) screened examples of these previously-engineered quadruplet-decoding tRNA^Pyl^ variants (Niu et al., 2013; Wang et al., 2014; Wang et al., 2016 N.; Chen et al., 2018) in HEK293 cells, and found that two of the tRNA variants (one evolved for mammalian cells, one with a simple quadruplet anticodon change) were suitable for use in ncAA implementation. Notably, engineered quadruplet tRNAs across all experiments were generally most effective only in the organism that were designed in.

A more recent investigation sought to improve different quadruplet *E. coli* tRNA suppression efficiencies through directed evolution (DeBenedictis et al., 2021) at the UAGA-codon, permitting multiplex decoding of four distinct quadruplet codons within a single reporter protein. It was found that mutations in the anticodon loop (positions 32 and 38), variable arm, central loop, D-stem, and acceptor stem improved quadruplet coding efficiency up to 80-fold. However, there was no clear consensus for these mutations. In a follow up experiment, focused libraries at positions 32, 37, and 38 further improved translation efficiency for different quadruplet codons in *E. coli* (DeBenedictis et al., 2022). Individual libraries for specific codons demonstrated enrichment for a specific nucleotide at one or more positions, suggesting significant covariance between the anticodon sequence and other regions of the anticodon arm. Additionally, there was a pronounced preference for base A37 across all codon libraries.

Quadruplet decoding has also been utilized to incorporate ncAAs in animals; to adapt quadruplet decoding ncAA incorporation in *C. elegans*, hybrid tRNAs containing the UCUA anticodon were created and demonstrated significant improvement in ncAA incorporation efficiency. They used *M. mazei* tRNA^Pyl^ variants already known to interact well with *C. elegans* translational machinery in triplet codon ncAA incorporation (Serfling et al., 2018; Davis et al., 2021) as a starting scaffold while overlaying anticodon arm modifications previously identified in (Niu et al., 2013; Wang N. et al., 2016).

With many mutational and structural studies conducted elucidating several anticodon arm engineering principles thus far, engineering quadruplet codons with sufficient translational fidelity is now more feasible than ever before, bringing us closer to a drastically expanded genetic code.

## 4 Improving peptide bond formation and peptidyl transfer

Aminoacylated tRNAs undergo innumerable interactions with translation factors and the ribosome on their journey to translating a polypeptide. After transport, initial acceptance, and codon-anticodon interactions, the P-site peptidyl-tRNA amino acid chain is transferred to the A-site aa-tRNA through a peptidyl transfer reaction. It is hypothesized that the ability of a ncAA to go through the peptidyl transfer (PT) reaction pathway is just as important—if not more important—than its ncAA’s affinity to EF-Tu (Katoh et al., 2017a).

Pro is a difficult ribosomal substrate during PT due to its rigid, bulky structure; and in this way, mimics ncAAs which are predominantly ill-suited for ribosomal translation. Pro directly slows down peptide bond formation compared to other AAs, with consecutive Pro residues leading to ribosomal stalling (Muto and Ito, 2008; Wohlgemuth et al., 2008; Pavlov et al., 2009; Doerfel et al., 2013; Peil et al., 2013; Ude et al., 2013; Starosta et al., 2014; Woolstenhulme et al., 2015; Melnikov et al., 2016a; Huter et al., 2017) and even protein truncation (Menninger, 1976; Cruz-Vera et al., 2004; Gonzalez de Valdivia and Isaksson, 2005; Muto and Ito, 2008). However, elongation factor P (EF-P) is an essential and well-conserved translation factor that alleviates ribosomal stalling caused by difficult ribosomal substrates like Pro and Gly (Katoh and Suga, 2018; Hummels and Kearns, 2020). EF-P binds on the ribosome between the E and P sites to interact with the P-site peptidyl-tRNA and stabilize its 3′ CCA end for favorable PTC positioning (Hummels and Kearns, 2020), which circumvents the inherently low amino acid reactivity and restores canonical transfer efficiency (Doerfel et al., 2015; Melnikov et al., 2016a).

EF-P specifically recognizes a unique nine nucleotide D-loop and stable 4 bp D-stem sequence observed in canonical tRNA^Pro^ (Blaha et al., 2009; Katoh et al., 2016). Thus, researchers have begun implementing the D-arm sequence of tRNA^Pro^ in engineering efforts to recruit EF-P and promote efficient PT for ncAAs (Figure 4). Many Pro-like ncAAs have since been attached to tRNA^Pro^ derivatives (Doerfel et al., 2015; Katoh et al., 2016), and were more efficiently incorporated in an EF-P-dependent manner. In another investigation, non-Pro-like ncAAs also significantly benefitted from this strategy when charged onto a tRNA^Pro^ derivative (Katoh et al., 2017a). Thus, optimizing tRNA structure for EF-P binding can improve ribosomal incorporation of a variety of ncAAs. Results in tRNA engineering efforts for efficient EF-P recruitment will vary according to the nascent peptide length (Gonzalez de Valdivia and Isaksson, 2005) and surrounding sequence context (Peil et al., 2013; Elgamal et al., 2014; Starosta et al., 2014). Generally, it was also shown that the more difficult a substrate is to ribosomally incorporate, the more it benefits from EF-P-dependent tRNA engineering. Further optimization of tRNA affinity for EF-P is tricky because the full mechanism of EF-P-mediated ncAA incorporation is unknown (Hummels and Kearns, 2020; Katoh et al., 2020). More intricate biochemical details on this process will likely elucidate a further comprehensive guide for this tRNA engineering strategy.

## 5 Improving multiple tRNA binding partner interactions simultaneously

### 5.1 tRNA^Pro1E2^ revolutionizes ribosomal ncAA incorporation

An interesting feature of tRNAs is their ability to be modularly engineered for simultaneous tuning of different translational processes. Such an idea was recently exploited to produce tRNA^Pro1E2^, a chimera between tRNA^Pro^ and tRNA^Glu^ (Katoh et al., 2017a) that allowed for simultaneous improvement of both EF-P and EF-Tu interactions. This tRNA transferred the T-stem and elements of the acceptor stem from tRNA^Glu^ (high natural EF-Tu affinity tRNA) onto the tRNA^Pro^ scaffold (optimized recruitment of EF-P) to synergistically improve the translational fidelity of difficult ribosomal substrates *in vitro* with optimized IF2, EF-G, EF-Tu, EF-Ts, and EF-G concentrations. In the presence of EF-P, tRNA^Pro1E2^ significantly outperformed other engineered tRNAs with similar high EF-Tu affinity (like tRNA^GluE2^) to incorporate d-AAs (Katoh et al., 2017a).

In light of this research, tRNA^Pro1E2^ has recently been used as an updated GCE scaffold tRNA to improve the installation of a staggering number of translation-incompatible substrates *in vitro* for a number of applications (Lee et al., 2020a; Lee et al., 2020b; Lee et al., 2021b.; Lee et al., 2022; Hammerling et al., 2020; Tsiamantas et al., 2020; Katoh and Suga, 2022b; Katoh and Suga, 2022a; Katoh and Suga, 2023b). As discussed above, this platform has been recently updated by adjusting the anticodon arm according to the anticodon sequence for further improving *in vitro* ncAA incorporation (Katoh and Suga, 2024), demonstrating the modular tunability of different tRNA regions.

### 5.2 Non-specific tRNA engineering with directed evolution

Directed evolution offers a strategy to engineer tRNAs by linking tRNA incorporation efficiency to an organism’s survival, helping to bypass the difficulties of rational design (Cobb et al., 2013; Miller et al., 2020; Wang et al., 2021). However, a common drawback of directed evolution is that it may be difficult to decode the results and extract general engineering principles.

In one notable example, compartmentalized partnered replication (CPR) (Abil et al., 2017), was used to co-evolve the *Saccharomyces cerevisiae* TrpRS:tRNA^sup^ pair for improved site-specific incorporation of the ncAA 5-hydroxy-l-tryptophan (5HTP) both *in vitro* and *in vivo* (Ellefson et al., 2014). Libraries targeting the anticodon stem, acceptor stem, or loop sequences (variable loop, D-loop, and one nucleotide in the T-loop) were generated independently. After 10 rounds of evolution, one tRNA variant (“40A”) demonstrated ∼12-fold better stop codon suppression than the original tRNA. The 40A variant contained one nucleotide change each in the D-loop, variable loop, and T-loop (U16G, G43U, U58G, respectively), highlighting the interesting solutions that directed evolution approaches often reveal.

Other examples of directed evolution are included in this review and detailed in other sections as they are often localized to specific tRNA regions. Nonetheless, broad directed evolution may serve to help implement extremely difficult ncAAs or optimize overall tRNA structure in ways that may not be feasible to rationally derive.

## 6 Translocation through the ribosome

After a new peptide bond is formed and the nascent polypeptide chain is transferred to the A-site, efficient translocation of the A- and P-site tRNAs to the P- and E-sites, respectively, must occur to mark a completed cycle of elongation. Recently, tRNA engineering efforts have sought to explore the modulation of this process.

### 6.1 Selenocysteine tRNA engineering for improved translocation

Great strides regarding *in vivo* Sec insertion have been made by engineering tRNA^UTu^, tRNA^UTuX^ tRNA^SecUx^, and tRNA^UTuT^ variants as previously discussed (Aldag et al., 2013; Miller et al., 2015; Thyer et al., 2015; Fan et al., 2018). However, Sec insertion efficiency and overall protein yields in these rewired Sec insertion pathways were still relatively low (Chung et al., 2022). EF-Tu only minimally binds tRNA with a 13 bp acceptor domain, even with a high-affinity antideterminant sequence (Rudinger et al., 1996). Therefore, exhaustive engineering efforts with tRNA^Sec^ and tRNA^Ser^ scaffolds could not reconcile the inherent discrepancy between the 13 bp acceptor domain necessary for SelA binding (but efficiently binds the elongation factor SelB (Fischer et al., 2016) and the 12 bp acceptor domain preferred for EF-Tu binding (Ohtsuki et al., 2005) in the rewired pathway.

Thus, a metagenomic search for EF-Tu-recognizing, tRNA^Sec^-like tRNAs revealed a uniquely structured class of tRNAs that demonstrated efficient Sec incorporation: allo-tRNAs (Mukai et al., 2017). They contain a longer D-stem (6 bp), smaller D-loop (4 bases), and a unique acceptor domain consisting of a 9 bp acceptor stem and 3 bp T-stem. One Ser-isoacceptor of interest, dubbed allo-tRNA^UTu1^, demonstrated efficient EF-Tu-mediated Sec installation when combined with *Aeromonas salmonicida* SelA (Mukai et al., 2018), which recognizes a 12-bp acceptor domain (Mukai et al., 2016). Further engineering of the tRNA^UTu1^ D-arm to improve SelA binding showed a significant improvement in Sec insertion. This tRNA and others (allo-tRNA^UTu2D^) have since been reliably used for downstream applications (Evans et al., 2021; Evans et al., 2024; Morosky et al., 2023).

However, as a non-native *E. coli* ribosome substrate, the suitability and processivity of these allo-tRNAs during protein synthesis on the bacterial ribosome was studied via single-molecule FRET and cryo-EM (Prabhakar et al., 2022). It was determined that tRNA^UTu1^ was able to induce on-par suppression efficiency and decoding time compared to native suppressor tRNA^supD^. However, single-molecule FRET analysis indicated that an overwhelming amount of ribosomal disassembly (64%) and low translational levels of processivity (21%) occurred after A-to P-site translocation. Structural determination of tRNA^UTu1^ in the bacterial ribosome determined that the long variable arm of tRNA^UTu1^ must undergo significant variable arm repositioning to avoid clashing with the ribosomal A-site finger (ASF) during translocation. The ASF is a region of the 23S rRNA that directly interacts with the A-site tRNA elbow, T-loop (Nguyen and Whitford, 2016), and amino acid during decoding to attenuate translocation and maintain overall translational fidelity (Yusupov et al., 2001; Komoda et al., 2006; Nguyen and Whitford, 2016; Nguyen et al., 2017). Specifically, interactions between bases C8 and A45 within the central loop were hypothesized to overly stabilize the variable arm and cause steric hindrance with the ASF (Figure 4). A single C8A mutation on tRNA^UTu1^ disrupted the variable arm stability and restored post-suppression codon survival *in vivo* to canonical efficiency (Prabhakar et al., 2022).

tRNA flexibility, especially stemming from the tRNA elbow (interactions between the D-arm, T-arm, and variable arm) clearly plays an important role in dictating the aa-tRNA’s journey through the ribosome (Frank et al., 2005; Zhou et al., 2014). More detailed research focused on the tRNA interactions through translocation and translation at large is emerging in prokaryotic systems (Ling and Ermolenko, 2016; Korostelev, 2022), which may aid in uncovering broad tRNA engineering principles to mediate this process.

### 6.2 The interconnected roles of EF-P and EF-G in translocation

Translocation is in large part mediated by the GTPase activity of elongation factor G (EF-G) in prokaryotes (Rodnina et al., 1997; Liu et al., 2011; Poulis et al., 2023) or eEF2 in eukaryotes (Kaul et al., 2011; Djumagulov et al., 2021; Milicevic et al., 2024). EF-G binds to the A-site to transfer deacylated P/E-site tRNA and peptidyl A/P-site tRNA to the E and P-sites, respectively. During states of slowed peptide bond formation and peptidyl transfer from inefficient substrates and/or excessive EF-G concentrations, EF-G is more likely to pre-maturely catalyze translocation before complete peptidyl transfer (mistranslocation). This can then induce drop-off of the aberrant P-site peptidyl tRNA from the ribosome, and in some cases, reinitiation of the aa-tRNA with active ribosomes to produce C- and N-terminally truncated proteins, respectively (Kang and Suga, 2011; Katoh et al., 2017a; Katoh et al., 2017b).

Both EF-G and the ribosome make important contacts with aa-tRNAs (Gao et al., 2009; Liu et al., 2011; Carbone et al., 2021), and thus engineering to improve these contacts presents itself as an avenue to mediate translational efficiency (Figure 4). However, simply maximizing an aa-tRNA’s EF-G affinity is not appropriate as it can lead to mistranslocation. Recent research (Tajima et al., 2022) approximates the “default” state of cellular protein synthesis as a state with high peptidyl transfer efficiency and low translocation frequency. This offers an initial conceptual framework for balancing these processes, the specifics of which are still unknown.

## 7 tRNA engineering in eukaryotic systems

Prokaryotic tRNAs have been easier to engineer than eukaryotes due to the relative simplicity of prokaryotic translation and a plethora of structural and mutagenic investigations mapping specific tRNA contacts with translation factors and the ribosome. Eukaryotic translation is still well-researched (Korostelev, 2022), though engineering specific sequences in tRNAs for enhanced translational outcomes in eukaryotes is less documented and has elucidated less guiding principles (Figure 4B). For example, specific bases in the T-stem and D-stem of tRNAs are known to impact binding with EF-Tu affinity and EF-P, respectively, with supporting structural determinations (discussed above). However, tRNA sequence-dependent affinity to equivalent eukaryotic factors eIF1A and eIF5A is considerably less understood. Regardless, research has still been aimed at optimizing tRNAs in various eukaryotic systems (Figure 4B). Current efforts have been focused on the Pyl and Sec insertion systems to improve their activities in eukaryotes.

### 7.1 Eukaryotic directed evolution

Effective aaRS:tRNA pairs from bacteria and archaea are often incorporated into higher eukaryotes because of inherent orthogonality, but this can lead to sub-optimal translational efficiency due to the presence of different translation machinery. Directed evolution methods have been established in *E. coli* to overcome this barrier, but an equivalent method for mammalian systems had not existed until recently (Jewel et al., 2023).

Such a directed evolution approach for mammalian systems, known as virus-assisted directed evolution (VADER), was recently developed (Jewel et al., 2023). Targeted evolution of the acceptor and T-stem regions for *Methanosarcineae mazei* tRNA^Pyl^, and the acceptor stem of *E. coli* tRNA^Tyr^, led to significant host-specific suppression efficiency improvement (Jewel et al., 2023). It was determined that the acceptor stem sequences made a significant impact on mammalian efficiency while the best T-stem sequences mirrored the wild-type sequence for tRNA^Pyl^ variants. Broadly, in tRNA^Pyl^ and tRNA^Tyr^ alike, the acceptor stems were significantly more G:C rich, with G residues preferentially on the 5′ side of the acceptor stem. However, understanding the reason for sequence preferences requires a better understanding of tRNA biology in mammalian cells. To begin uncovering the basis of these improvements, the authors compared the activity in *E. coli* of their best-performing tRNA^Pyl^ (A2.1) with wild-type *M. mazei* tRNA^Pyl^. It was shown that while A2.1 performed better in HEK293T, these improvements had no significant effect on its activity in *E. coli*. This emphasizes that the protein synthesis machinery differs between hosts, and tRNA sequences improved in one host cannot necessarily be transferred to others. Follow-up research identified a tRNA^Pyl^ variant, PyOtR, with markedly improved activity over A2.1 in mammalian cells (Jewel et al., 2024). PyOtR contained a similarly GC-rich acceptor stem, with a bias for 5′ G residues, but also demonstrated an essential G5:U68 wobble pair—usually detrimental in tRNA acceptor stems (Santoro et al., 2003). Another study also recently used this technology to evolve the acceptor stem of *E.* coli tRNA^Leu^ for ncAA incorporation in eukaryotes (Huang et al., 2024). *E. coli* tRNA^Leu^ is a well-known platform for ncAA incorporation in eukaryotic systems, but typically suffered from low activity (Chin, 2017; Italia et al., 2017; Young and Schultz, 2018). The two best-performing mutants from this study contained G1:C72 and U6:G67 in the acceptor stem which led to substantial improvement in the incorporating ncAAs. The basis behind why these mutations improve translational fidelity in mammalian systems is still unclear, but the ability to generate and screen large tRNA libraries will likely guide research efforts to uncover key eukaryotic interactions and refined tRNA engineering principles.

### 7.2 tRNA^Pyl^

Archaeal tRNA^Pyl^ is significantly divergent from canonical tRNAs, containing a smaller D-loop, variable loop, and longer anticodon stem. This unique structure confers orthogonality in other domains of life which makes them a scaffold for GCE in bacteria and eukaryotes alike (Wan et al., 2014; Tharp et al., 2017; Krahn et al., 2024). However, implementing the Pyl system into mammalian cells had generally resulted in modest protein yields (Serfling et al., 2018). It was known from previous research with tRNA^Leu^ that replacing anticodon stem region G:U wobble pairs with Watson-Crick base pairs improved tRNA activity (Anderson and Schultz, 2003), so this same idea was applied to modestly improve the translational fidelity of *M. mazei* tRNA^Pyl^ in both *E. coli* (Chatterjee et al., 2013) and HEK293T cells (Schmied et al., 2014).

More recent work screened small collections of rationally designed *M. mazei* tRNA^Pyl^ variants to improve recognition by endogenous mammalian translation machinery. This was done by incorporating conserved human tRNA sequence patterns (Jühling et al., 2009; Giegé and Frugier, 2013; Suzuki et al., 2020) from mammalian tRNAs. These variants yielded more robust incorporation of one or several ncAAs into a G-protein coupled receptor in mammalian cells although the exact mechanism(s) of action are unknown. The top mutants demonstrated considerable sequence variability, but there were still some hints at potential eukaryotic tRNA engineering principles. The top mutants contained a specific, stabilizing G19:C56 tertiary interaction (“hinge”) between the D- and T-loops broadly conserved in tRNAs (Serfling et al., 2018; Berg and Brandl, 2021). Additionally, base pair G7:C66 (opposed to wild-type C7:G66) improved tRNA efficiency, a similarity found in bacteria (Guo et al., 2009; Fan et al., 2015). This base pair is known to impact aa-tRNA binding affinity to EF-Tu, hinting at the idea that tRNAs interact with EF-Tu in a similar way as eIF1A. This is further supported by evolution of a G7:C66 sequence in efficient tRNA^Pyl^ variants from a directed evolution experiment in mammalian cells (Zhao et al., 2021). These experiments importantly demonstrate the ability of tRNAs to be adapted for use in organisms with heterologous protein synthesis machinery, although the details of the specific eukaryotic protein synthesis mechanisms and tRNA engineering principles are only beginning to be determined.

### 7.3 tRNAs for inserting Sec

While many organisms contain Sec machinery, this machinery is widely absent in the Fungi kingdom (Mariotti et al., 2019). For the Pyl system, tRNA^Pyl^ has been modified to resemble mammalian tRNAs to improve interactions with mammalian translation machinery (discussed above). Recent Sec research used the opposite approach to produce Sec-containing proteins in yeast; *S. cerevisiae* tRNA^Ser^ isoacceptor (tRNA-Ser-AGA-1-1, (Chan and Lowe, 2016) was modified to resemble a Sec tRNA. Through this strategy, a synthetic tRNA was generated that is compatible with yeast translation machinery and capable of Sec biosynthesis when artificially provided with the additional machinery (Hoffman et al., 2023). Many of these tRNA mutations were designed primarily to increase the efficiency of aminoacylation with Sec (i.e., tuning the tRNA sequence for binding to yeast SerRS and heterologous *A. salmonicida* SelA). However, it was shown that a G59U mutation (critical for maintaining 3D structure), was important for Sec insertion, and this engineered tRNA was capable of eEF1A-mediated delivery to the ribosome. Future structural and mutational research on this system will almost certainly uncover new rational tRNA engineering principles to improve translational fidelity in yeast.

## 8 Initiator tRNA engineering

Translation begins with some form of a methionine amino acid in all domains of life (Bhattacharyya and Varshney, 2016). A protein’s N-terminus is also integral for regulating co-translational modifications and protein half-lives (Varshavsky, 2011), therefore important for site-specific amino acid implementation. Initiation with non-canonical initiation substrates can often induce truncated proteins lacking the first amino acid (Katoh and Suga, 2023a); however, mis-charging of *E. coli* tRNA^fMet^ with other amino acids has regularly demonstrated diverse ncAA incorporation at the N-terminus of proteins without extensive tRNA engineering (Chattapadhyay et al., 1990; Goto et al., 2008a; Goto et al., 2008b; Rogers et al., 2018; Ad et al., 2019; Tharp et al., 2020b). This simply requires utilizing UAG-recognizing initiator tRNAs; otherwise, an engineered AUG-recognizing initiator tRNA will compete with natural, Met-inserting initiator tRNAs at AUG start codons, resulting in misincorporation. (Tharp et al., 2020b).

In bacteria, tRNA^fMet^ is charged with formylated methionine (fMet) as a positive determinant for binding IF2 and a negative determinant for binding EF-Tu (Faulhammer and Joshi, 1987; Wang et al., 2015; Shah et al., 2019). A 1:72 bp mismatch in *E. coli* confers the conserved identity element for methionine formylation by methionyl-tRNA transformylase, exclusion of the tRNA from the ribosomal A-site, and resistance to peptidyl-tRNA hydrolase (Mandal et al., 1996). The anticodon stem also contains three G:C base pairs (G29:C41, G30:C40, and G31:C39) critical for P-site binding in both prokaryotic and eukaryotic initiator tRNAs (Seong and RajBhandary, 1987; Mandal et al., 1996; Rasmussen et al., 2009). Crucially, these two features can be transplanted to an elongator tRNA to convert it into an initiator tRNA (Varshney et al., 1993). Also important for formylation are base pairs 2:71 and 3:70 in the acceptor stem, and 11:24 in the D-stem (RajBhandary, 1994; Rasmussen et al., 2009; Lee B. S. et al., 2021). Lastly, G29:C41 and G30:C40 base pairs in the anticodon stem are required for efficient protein synthesis (Mandal et al., 1996). In eukaryotes, the initiator tRNA is charged with methionine (instead of fMet), although the tRNA structure differs from canonical tRNA^Met^. Current information also shows that the initiator tRNA contains a specific A1:U72 base pair required for eIF2 binding (Rasmussen et al., 2009).

**FIGURE 6:**
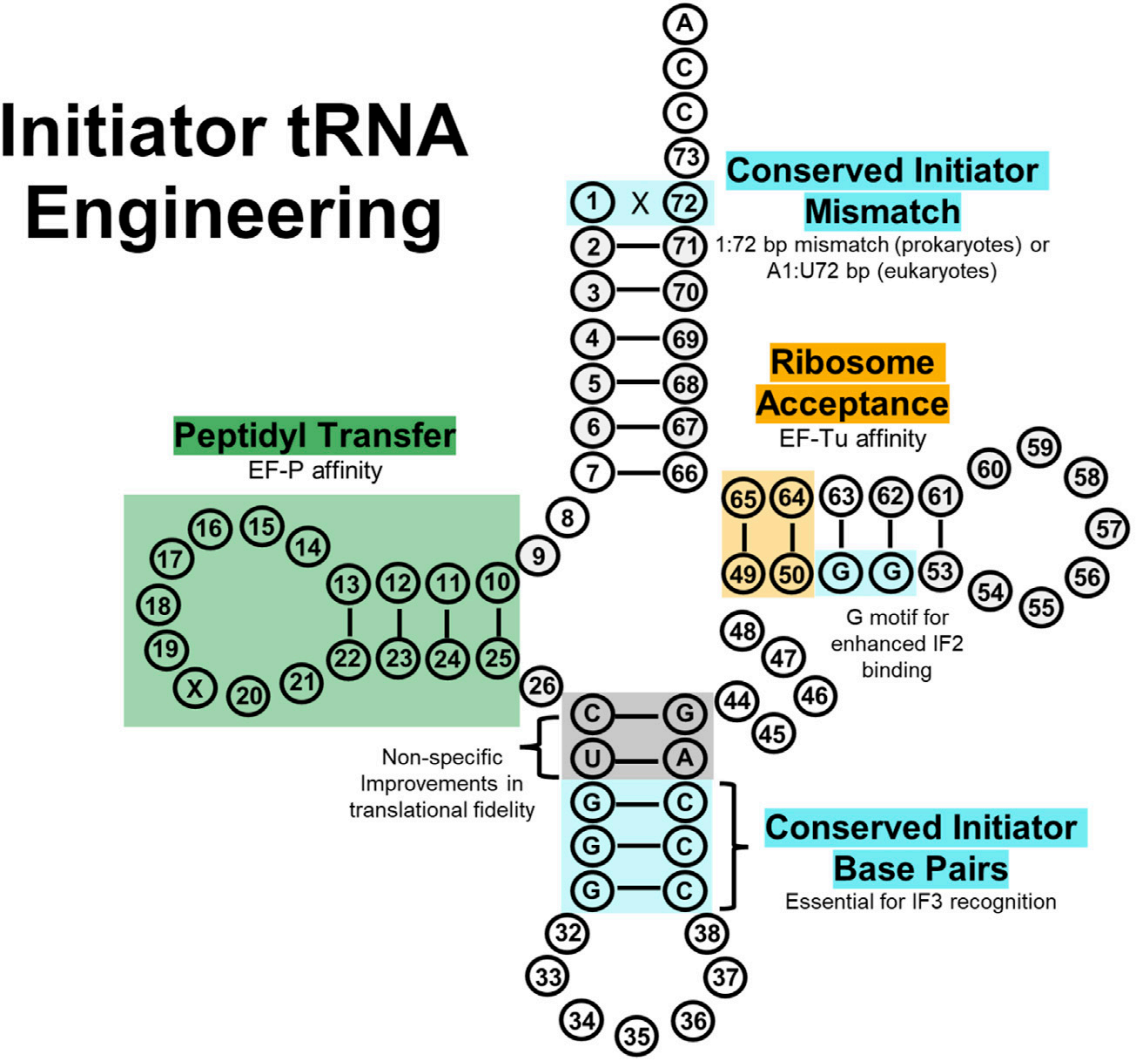
Initiator tRNA engineering sites. Initiator tRNAs contain unique sequence features (light blue) to encourage proper aminoacylation and binding to the ribosome P-site, as opposed to elongator tRNAs which bind the A-site. Sequences governing EF-Tu (green) and EF-P (orange) affinity have been carried over from elongator tRNA engineering research to improve initiator tRNA fidelity. Additionally, directed evolution has determined some non-specific improvements (grey) in the anticodon stem that further improve fidelity, including a G51-G52 motif in the T-stem that enhances IF2 binding to help position initiator tRNAs in the ribosomal P-site.

tRNA engineering efforts for enhancing eukaryotic initiation have not yet been shown, but recent research has explored prokaryotic initiator tRNA engineering (Figure 6). Non-canonical initiation in prokaryotes has been shown both *in vitro* and *in vivo*, likely paving the way for future eukaryotic experimentation. One concern with incorporating ncAAs at the N-terminus is drop-off-reinitiation, where the aminoacyl initiator tRNA drops off from the ribosome and then translation is reinitiated with the second aa-tRNA in the A-site, resulting in an N-terminally truncated protein (Katoh and Suga, 2023a). Thus, tRNA engineering has been utilized to enhance initiator aa-tRNA activity in the ribosome. Recently, a bacterial initiator tRNA (tRNA^fMet2^) was engineered for *in vitro* incorporation of various ncAAs by increasing its affinity for EF-P while retaining core initiator tRNA function (Katoh and Suga, 2023c). EF-P functions in initiation to catalyze the peptide bond between the first and second amino acid (Blaha et al., 2009), although its potential role in improving translation initiation with ncAAs was unexplored. IF3 also functions in initiation, recognizing and positioning initiator tRNA into the P-site for start codon recognition (Hussain et al., 2016; Kaledhonkar et al., 2019). From this knowledge, a chimeric tRNA (tRNA^iniP^) was engineered from canonical initiator tRNA^fMet2^ and tRNA^Pro1^ (previously engineered for improved EF-P binding (Katoh et al., 2016) while retaining three G:C base pairs in the anticodon stem (G29:C41, G30:C40, and G31:C39) for IF3 recognition in initiation (Katoh and Suga, 2023c). tRNA^iniP^ contained other nucleotides from tRNA^Pro1^ (the D-arm motif for EF-P recognition, base pairs 27:43 and 28:42 in the anticodon stem, base pairs 49:65 and 50:64 in the T-stem, and nucleotide G46) as a result of screening different combinations of these chimeric tRNAs. The D-arm conferred EF-P affinity and the T-stem mutations likely fine-tuned EF-Tu affinity for improved translational fidelity, but the specific functions of the variable arm and anticodon stem mutations are not entirely obvious. Furthermore, the concentrations of IF3, EF-G, ribosome recycling factor, and sequences of the codon and SD were all optimized to help reduce drop-off-reinitiation. These mutations led to tRNA^iniP^ demonstrating nearly 1,000-fold improvement in peptide expression with a non-canonical initiation substrate compared to a conventional initiator tRNA (Katoh and Suga, 2023c).

Two different strategies have been applied to adapt the *Mj* tRNA^Tyr^:TyrRS system for translation initiation: i) engineering initiator tRNAs for *Mj* TyrRS recognition while retaining initiator tRNA sequence motifs (Tharp et al., 2020a; 2021a), and ii) repurposing elongator *Mj* tRNA^Tyr^ as an initiator tRNA (Lee B. S. et al., 2021).

The first strategy modified *E. coli* initiator tRNA^fMet^ for recognition by *Mj* TyrRS while retaining initiator tRNA sequence motifs to orthogonally incorporate diverse ncAAs *in vivo* (Tharp et al., 2020a). Nucleotide A73 and the C1:G72 base pair are sufficient for *Mj* TyrRS recognition and were transplanted into *E. coli* tRNA^fMet^ (resulting in *i*tRNA^Ty2^). Using *Mj* TyrRS and TyrRS variants, 16 different ncAAs were encoded during initiation at the UAG nonsense codon *in vivo* for the first time. Interestingly, follow-up work demonstrated an *i*tRNA^Ty2^ mutant (recoded to decode UAU codons) can selectively install ncAAs at the artificial UAU initiation codon while canonical machinery predominantly implements Tyr at repurposed elongator UAU codons (sense codon reassignment) (Tharp et al., 2021a). This importantly demonstrates the inherent orthogonality between initiator and elongator tRNAs, although it is not always exclusive (Tharp et al., 2021a). In the second strategy, *Mj* tRNA^Tyr^ was converted into an amber-suppressing initiator tRNA using *E. coli* initiator tRNA identity elements (Lee B. S. et al., 2021). Here, *Mj* tRNA^Tyr^_CUA_ was engineered to contain a C1:A72 base pair mismatch (critical for methionyl-tRNA transformylase interactions), three G:C pairs in the anticodon stem (for binding the 30S P-site), and a G51-G52 motif for enhanced IF2 binding (Rasmussen et al., 2009). This resulted in *Mj*-itRNA-2, which, when combined with overexpression of methionyl-tRNA transformylase, efficiently encoded a ncAA into the amber start codon but not in identical elongation amber codons (Lee B. S. et al., 2021).

## 9 Therapeutic applications of tRNA engineering

The aberrant generation of a stop codon in a gene is the cause of nearly 11% of all human genetic disorders (Mort et al., 2008). As such, recent research has sought to develop safe and efficient suppressor tRNAs to readthrough pathogenic stop codons and rescue disrupted mRNAs and truncated proteins (Porter et al., 2021; Awawdeh et al., 2024; Coller and Ignatova, 2024). tRNA suppression therapy is at an early stage of development, with most research at the pre-clinical phase. Still, tRNA therapies compliment recent advances in the broader field of RNA therapeutics (like mRNA-based vaccines) and synthetic biology regarding GCE. These tRNA therapies give rise to unique medical benefits and inhibitions compared to the extensively researched genetic engineering toolbox of various CRISPR- and CRISPR-adjacent platforms (Terns, 2018; Petraitytė et al., 2021; Chehelgerdi et al., 2024). Certainly, engineering therapeutic suppressor tRNAs for proper efficiency during elongation (or initiation) is a necessity and evidenced by several recent start-up companies developing tRNA therapeutics (Dolgin, 2022; Anastassiadis and Köhrer, 2023). Although outside the scope of this review, an excellent recent review is provided which details tRNA therapeutics and delivery (Ward et al., 2024).

The simplest and most common approach for alleviating PTCs is to supply individuals with a natural suppressor tRNA containing a repurposed anticodon to readthrough the PTC. This allows for the correct amino acid to be inserted and alleviate translation termination. Recently, a high-throughput, cell-based assay has been developed to screen such anticodon-engineered tRNAs for efficient suppression: ACE-tRNA (Lueck et al., 2019; Ko et al., 2022; Lin and Glatt, 2022). In this way, effective tRNAs must be sufficiently recognized by the protein synthesis machinery. However, the efficiency of these identified anticodon-engineered suppressor tRNAs was modest and may only be applicable to diseases with a low therapeutic threshold (Zhou et al., 2012; Bordeira-Carriço et al., 2014; Lueck et al., 2019; Porter et al., 2021; Coller and Ignatova, 2024). This is because i) a significant amount of tRNA isoacceptors lose activity when their anticodon arms are altered (Lueck et al., 2019), ii) correct positioning within the decoding center is compromised (Westhof et al., 2022) and cannot outcompete the nonsense-mediated mRNA decay pathway (Albers et al., 2023), and iii) anticodon-engineered tRNAs can be prone to incorporating the wrong amino acid (Kaiser et al., 2020).

These results still suggest that anticodon-modified tRNAs have some utility, and combining it with modifications to other regions of the tRNA can improve translational fidelity (Ogawa et al., 2011; Ogawa et al., 2012). Thus, other research has focused on holistically designing therapeutic suppressor tRNAs for improved translational efficiency. Bacterial tRNAs have been repurposed to efficiently and exclusively install Ala at a UGA PTC in *E. coli* (Albers et al., 2021). These tRNAs were designed *in silico* and programmed to have the suppressor anticodon and maintain key tertiary interactions for recognition by AlaRS, while varying the other nucleotides. However, these *de novo* tRNAs only displayed significant *in vivo* suppression when further engineered to contain an optimized T-stem for EF-Tu binding. In this case, the tRNA^Glu^ T-stem was transplanted to accommodate the destabilizing effect of Ala with EF-Tu. Testing of further variants using the D-arm from tRNA^Pro^ for enhanced EF-P affinity displayed modest impact in a variant-specific manner. Furthermore, modifications to lengthen the variable arm decreased suppression. In some studies, a long variable arm is associated with higher suppression, however the sequence of the tRNA core also plays a role in the efficiency of mRNA translation (Geslain and Pan, 2010; Chung and Krahn, 2022; Prabhakar et al., 2022). Structural determination of the most effective variant (t1A3T2) in the ribosome also suggested that engineered suppressor tRNAs still undergo a similar decoding mechanism as canonical sense codons, further confirming on a structural level that research surrounding canonical sense codon translation can be applied to further develop tRNA therapeutics.

Follow-up work used similar engineering principles to convert native human tRNAs into efficient suppressor tRNAs in mammalian cells for treating readthrough of the cystic fibrosis transmembrane conductance regulator gene *CFTR* (Albers et al., 2023). Three families of human tRNAs were engineered which resulted in considerably improved *CFTR* PTC suppression in mammalian cell lines and patient-derived human nasal epithelial cells at different stop codons. Similar suppression improvement was also shown when tRNAs were encapsulated in liquid nanoparticles (LNPs) and delivered to mice. The researchers importantly designed these tRNAs based on known bacterial tRNA engineering principles; the T-stem was modified for improved interactions with eEF1A (Andersen et al., 2000; Schrader et al., 2011) and the anticodon arm was modified to supplement decoding (Yarus et al., 1986; Nguyen et al., 2020). Importantly, suppression efficacy was dependent on both the stop codon sequence and sequence context surrounding the PTCs. Optimizing tRNAs for sequence context (in both therapeutic and synthetic biology applications) is an important variable that must be considered in tRNA design. As such, it has been determined that PTC-upstream translation velocity (using Ribo-seq data) can be modeled to accurately predict suppression fidelity at different PTCs when designing engineered suppressor tRNAs (Bharti et al., 2024).

Similar strategies may also be applied for missense mutations which account for a large portion of pathogenic human diseases, and are seemingly used naturally to compensate for environmental stress in bacteria (Schuntermann et al., 2023). Such missense-correcting tRNAs are charged with an amino acid but programmed to read another amino acid’s cognate anticodon. Missense-correcting-tRNAs have been recently explored in mammalian cells and are likely to benefit from further engineering to improve translational fidelity (Bily et al., 2024; Hou et al., 2024).

## 10 Discussion

tRNAs play a role in every step of translation, often by directly interacting with the ribosome and other translation factors. As such, substantial research has mapped the interactions of tRNA sequence segments and even post-transcriptional modifications with these binding partners. Combined, this results in a robust “map” that can guide tRNA engineering efforts to mediate each of the key steps in translation, or several steps in parallel due to the modularity of different tRNA regions. With significant advances in structural and computational biology, a greater mechanistic understanding of the various roles of tRNA in translation will certainly uncover more rational engineering principles. Artificial intelligence and machine learning technologies in academia (Coller and Ignatova, 2024) and industry (e.g., https://www.alltrna.com/) alike are just beginning to trailblaze a new frontier in tRNA engineering research by predicting biomolecular structures and interactions to simulate and guide tRNA engineering efforts. Specifically, the recent release of AlphaFold3 newly allows for the prediction of protein-nucleic acid interactions which will certainly progress tRNA engineering research by offering another tool for illuminating tRNA interactions throughout protein synthesis (Abramson et al., 2024). Additionally, powerful directed evolution approaches adapted to prokaryotic and eukaryotic systems alike have dramatically improved the translational fidelity of tRNAs, bypassing the complexity of rational design. tRNA engineering has thus shined as a robust tool for applications in basic research, synthetic biology, and therapeutics.

## References

[B1] Abil Z. Ellefson J. W. Gollihar J. D. Watkins E. Ellington A. D. (2017). Compartmentalized partnered replication for the directed evolution of genetic parts and circuits. Nat. Protoc. 12, 2493–2512. 10.1038/nprot.2017.119 29120463 PMC6053311

[B2] Abramson J. Adler J. Dunger J. Evans R. Green T. Pritzel A. (2024). Accurate structure prediction of biomolecular interactions with AlphaFold 3. Nature, 1–3. 10.1038/s41586-024-07487-w PMC11168924 38718835

[B3] Achenbach J. Jahnz M. Bethge L. Paal K. Jung M. Schuster M. (2015). Outwitting EF-Tu and the ribosome: translation with d-amino acids. Nucleic Acids Res. 43, 5687–5698. 10.1093/nar/gkv566 26026160 PMC4499158

[B4] Ad O. Hoffman K. S. Cairns A. G. Featherston A. L. Miller S. J. Söll D. (2019). Translation of diverse aramid- and 1,3-dicarbonyl-peptides by wild type ribosomes *in vitro* . ACS Cent. Sci. 5, 1289–1294. 10.1021/acscentsci.9b00460 31403077 PMC6661870

[B5] Agris P. F. (1991). Wobble position modified nucleosides evolved to select transfer RNA codon recognition: a modified-wobble hypothesis. Biochimie 73, 1345–1349. 10.1016/0300-9084(91)90163-U 1799628

[B6] Agris P. F. (2004). Decoding the genome: a modified view. Nucleic Acids Res. 32, 223–238. 10.1093/nar/gkh185 14715921 PMC384350

[B7] Agris P. F. (2008). Bringing order to translation: the contributions of transfer RNA anticodon‐domain modifications. EMBO Rep. 9, 629–635. 10.1038/embor.2008.104 18552770 PMC2475317

[B8] Agris P. F. Narendran A. Sarachan K. Väre V. Y. P. Eruysal E. (2017). “Chapter one - the importance of being modified: the role of RNA modifications in translational fidelity,” in The enzymes. Editor Chanfreau G. F. (Academic Press), 1–50. 10.1016/bs.enz.2017.03.005 PMC8118379 28601219

[B9] Albers S. Allen E. C. Bharti N. Davyt M. Joshi D. Perez-Garcia C. G. (2023). Engineered tRNAs suppress nonsense mutations in cells and *in vivo* . Nature 618, 842–848. 10.1038/s41586-023-06133-1 37258671 PMC10284701

[B10] Albers S. Beckert B. Matthies M. C. Mandava C. S. Schuster R. Seuring C. (2021). Repurposing tRNAs for nonsense suppression. Nat. Commun. 12, 3850. 10.1038/s41467-021-24076-x 34158503 PMC8219837

[B11] Aldag C. Bröcker M. J. Hohn M. J. Prat L. Hammond G. Plummer A. (2013). Rewiring translation for elongation factor tu-dependent selenocysteine incorporation. Angew. Chem. Int. Ed. 52, 1441–1445. 10.1002/anie.201207567 PMC3776052 23193031

[B12] Anastassiadis T. Köhrer C. (2023). Ushering in the era of tRNA medicines. J. Biol. Chem. 299, 105246. 10.1016/j.jbc.2023.105246 37703991 PMC10583094

[B13] Andersen G. R. Pedersen L. Valente L. Chatterjee I. Kinzy T. G. Kjeldgaard M. (2000). Structural basis for nucleotide exchange and competition with tRNA in the yeast elongation factor complex eEF1A:eEF1B**α** . Mol. Cell 6, 1261–1266. 10.1016/s1097-2765(00)00122-2 11106763

[B14] Anderson J. C. Schultz P. G. (2003). Adaptation of an orthogonal archaeal leucyl-tRNA and synthetase pair for four-base, amber, and opal suppression. Biochemistry 42, 9598–9608. 10.1021/bi034550w 12911301

[B15] Anderson J. C. Wu N. Santoro S. W. Lakshman V. King D. S. Schultz P. G. (2004). An expanded genetic code with a functional quadruplet codon. Proc. Natl. Acad. Sci. U. S. A. 101, 7566–7571. 10.1073/pnas.0401517101 15138302 PMC419646

[B16] Arnér E. S. J. (2010). Selenoproteins-what unique properties can arise with selenocysteine in place of cysteine? Exp. Cell Res. 316, 1296–1303. 10.1016/j.yexcr.2010.02.032 20206159

[B17] Asahara H. Uhlenbeck O. C. (2002). The tRNA specificity of *Thermus thermophilus* EF-Tu. Proc. Natl. Acad. Sci. U. S. A. 99, 3499–3504. 10.1073/pnas.052028599 11891293 PMC122552

[B18] Asahara H. Uhlenbeck O. C. (2005). Predicting the binding affinities of misacylated tRNAs for *Thermus thermophilus* EF-Tu·GTP. Biochemistry 44, 11254–11261. 10.1021/bi050204y 16101309

[B19] Atkins J. F. Björk G. R. (2009). A gripping tale of ribosomal frameshifting: extragenic suppressors of frameshift mutations spotlight P-site realignment. Microbiol. Mol. Biol. Rev. 73, 178–210. 10.1128/mmbr.00010-08 19258537 PMC2650885

[B20] Auffinger P. Westhof E. (1999). Singly and bifurcated hydrogen-bonded base-pairs in tRNA anticodon hairpins and ribozymes. J. Mol. Biol. 292, 467–483. 10.1006/jmbi.1999.3080 10497015

[B21] Auffinger P. Westhof E. (2001). An extended structural signature for the tRNA anticodon loop. RNA 7, 334–341. 10.1017/S1355838201002382 11333014 PMC1370090

[B22] Awawdeh A. Radecki A. A. Vargas-Rodriguez O. (2024). Suppressor tRNAs at the interface of genetic code expansion and medicine. Front. Genet. 15, 1420331. 10.3389/fgene.2024.1420331 38798701 PMC11116698

[B23] Baldridge K. C. Jora M. Maranhao A. C. Quick M. M. Addepalli B. Brodbelt J. S. (2018). Directed evolution of heterologous tRNAs leads to reduced dependence on post-transcriptional modifications. ACS Synth. Biol. 7, 1315–1327. 10.1021/acssynbio.7b00421 29694026

[B24] Baron C. Böck A. (1991). The length of the aminoacyl-acceptor stem of the selenocysteine-specific tRNA^Sec^ of *Escherichia coli* is the determinant for binding to elongation factors SELB or Tu. J. Biol. Chem. 266, 20375–20379. 10.1016/S0021-9258(18)54933-4 1939093

[B25] Beattie A. T. Dunkelmann D. L. Chin J. W. (2023). Quintuply orthogonal pyrrolysyl-tRNA synthetase/tRNA^Pyl^ pairs. Nat. Chem. 15, 948–959. 10.1038/s41557-023-01232-y 37322102 PMC7615293

[B26] Ben-Shem A. Garreau de Loubresse N. Melnikov S. Jenner L. Yusupova G. Yusupov M. (2011). The structure of the eukaryotic ribosome at 3.0 Å resolution. Science 334, 1524–1529. 10.1126/science.1212642 22096102

[B27] Berg M. D. Brandl C. J. (2021). Transfer RNAs: diversity in form and function. RNA Biol. 18, 316–339. 10.1080/15476286.2020.1809197 32900285 PMC7954030

[B28] Bharti N. Santos L. Davyt M. Behrmann S. Eichholtz M. Jimenez-Sanchez A. (2024). Translation velocity determines the efficacy of engineered suppressor tRNAs on pathogenic nonsense mutations. Nat. Commun. 15, 2957. 10.1038/s41467-024-47258-9 38580646 PMC10997658

[B29] Bhattacharyya S. Varshney U. (2016). Evolution of initiator tRNAs and selection of methionine as the initiating amino acid. RNA Biol. 13, 810–819. 10.1080/15476286.2016.1195943 27322343 PMC5013993

[B30] Biela A. Hammermeister A. Kaczmarczyk I. Walczak M. Koziej L. Lin T.-Y. (2023). The diverse structural modes of tRNA binding and recognition. J. Biol. Chem. 299, 104966. 10.1016/j.jbc.2023.104966 37380076 PMC10424219

[B31] Bily T. M. I. Heinemann I. U. O’Donoghue P. (2024). Missense suppressor tRNA therapeutics correct disease-causing alleles by misreading the genetic code. Mol. Ther. 32, 273–274. 10.1016/j.ymthe.2024.01.001 38219738 PMC10861964

[B32] Blackmond D. G. (2010). The origin of biological homochirality. Cold Spring Harb. Perspect. Biol. 2, a002147. 10.1101/cshperspect.a002147 20452962 PMC2857173

[B33] Blaha G. Stanley R. E. Steitz T. A. (2009). Formation of the first peptide bond: the structure of EF-P Bound to the 70S Ribosome. Science 325, 966–970. 10.1126/science.1175800 19696344 PMC3296453

[B34] Blanchet S. Ranjan N. (2022). Translation phases in eukaryotes. Methods Mol. Biol. Clifton N. J. 2533, 217–228. 10.1007/978-1-0716-2501-9_13 PMC9761538 35796991

[B35] Bloom-Ackermann Z. Navon S. Gingold H. Towers R. Pilpel Y. Dahan O. (2014). A comprehensive tRNA deletion library unravels the genetic architecture of the tRNA pool. PLOS Genet. 10, e1004084. 10.1371/journal.pgen.1004084 24453985 PMC3894157

[B36] Bolton P. H. Kearns D. R. (1975). NMR evidence for common tertiary structure base pairs in yeast and *E. coli* tRNA. Nature 255, 347–349. 10.1038/255347a0 1093043

[B37] Bordeira-Carriço R. Ferreira D. Mateus D. D. Pinheiro H. Pêgo A. P. Santos M. A. S. (2014). Rescue of wild-type E-cadherin expression from nonsense-mutated cancer cells by a suppressor-tRNA. Eur. J. Hum. Genet. EJHG 22, 1085–1092. 10.1038/ejhg.2013.292 24424122 PMC4135406

[B38] Bossi L. Smith D. M. (1984). Suppressor sufJ: a novel type of tRNA mutant that induces translational frameshifting. Proc. Natl. Acad. Sci. 81, 6105–6109. 10.1073/pnas.81.19.6105 6091135 PMC391868

[B39] Brito Querido J. Díaz-López I. Ramakrishnan V. (2024). The molecular basis of translation initiation and its regulation in eukaryotes. Nat. Rev. Mol. Cell Biol. 25, 168–186. 10.1038/s41580-023-00624-9 38052923

[B40] Bubunenko M. G. Kireeva M. L. Gudkov A. T. (1992). Novel data on interactions of elongation factor ts. Biochimie 74, 419–425. 10.1016/0300-9084(92)90081-O 1637866

[B41] Burnett B. J. Altman R. B. Ferguson A. Wasserman M. R. Zhou Z. Blanchard S. C. (2014). Direct evidence of an elongation factor-Tu/Ts·GTP·Aminoacyl-tRNA quaternary complex. J. Biol. Chem. 289, 23917–23927. 10.1074/jbc.M114.583385 24990941 PMC4156062

[B42] Burnett B. J. Altman R. B. Ferrao R. Alejo J. L. Kaur N. Kanji J. (2013). Elongation factor ts directly facilitates the formation and disassembly of the *Escherichia coli* elongation factor tu·GTP·aminoacyl-tRNA ternary complex. J. Biol. Chem. 288, 13917–13928. 10.1074/jbc.M113.460014 23539628 PMC3650427

[B43] Cantara W. A. Bilbille Y. Kim J. Kaiser R. Leszczyńska G. Malkiewicz A. (2012). Modifications modulate anticodon loop dynamics and codon recognition of *E. coli* tRNA^Arg1,2^ . J. Mol. Biol. 416, 579–597. 10.1016/j.jmb.2011.12.054 22240457

[B44] Cantara W. A. Crain P. F. Rozenski J. McCloskey J. A. Harris K. A. Zhang X. (2011). The RNA modification database, RNAMDB: 2011 update. Nucleic Acids Res. 39, D195–D201. 10.1093/nar/gkq1028 21071406 PMC3013656

[B45] Cappannini A. Ray A. Purta E. Mukherjee S. Boccaletto P. Moafinejad S. N. (2024). MODOMICS: a database of RNA modifications and related information. 2023 update. Nucleic Acids Res. 52, D239–D244. 10.1093/nar/gkad1083 38015436 PMC10767930

[B46] Carbone C. E. Loveland A. B. Gamper H. B. Hou Y.-M. Demo G. Korostelev A. A. (2021). Time-resolved cryo-EM visualizes ribosomal translocation with EF-G and GTP. Nat. Commun. 12, 7236. 10.1038/s41467-021-27415-0 34903725 PMC8668904

[B47] Cervettini D. Tang S. Fried S. D. Willis J. C. W. Funke L. F. H. Colwell L. J. (2020). Rapid discovery and evolution of orthogonal aminoacyl-tRNA synthetase–tRNA pairs. Nat. Biotechnol. 38, 989–999. 10.1038/s41587-020-0479-2 32284585 PMC7116527

[B48] Chan P. P. Lowe T. M. (2016). GtRNAdb 2.0: an expanded database of transfer RNA genes identified in complete and draft genomes. Nucleic Acids Res. 44, D184–D189. 10.1093/nar/gkv1309 26673694 PMC4702915

[B49] Chattapadhyay R. Pelka H. Schulman L. H. (1990). Initiation of *in vivo* protein synthesis with non-methionine amino acids. Biochemistry 29, 4263–4268. 10.1021/bi00470a001 2112406

[B50] Chatterjee A. Lajoie M. J. Xiao H. Church G. M. Schultz P. G. (2014). A bacterial strain with a unique quadruplet codon specifying non-native amino acids. ChemBioChem 15, 1782–1786. 10.1002/cbic.201402104 24867343

[B51] Chatterjee A. Sun S. B. Furman J. L. Xiao H. Schultz P. G. (2013). A versatile platform for single- and multiple-unnatural amino acid mutagenesis in *Escherichia coli* . Biochemistry 52, 1828–1837. 10.1021/bi4000244 23379331 PMC3855549

[B52] Chatterjee A. Xiao H. Schultz P. G. (2012). Evolution of multiple, mutually orthogonal prolyl-tRNA synthetase/tRNA pairs for unnatural amino acid mutagenesis in *Escherichia coli* . Proc. Natl. Acad. Sci. 109, 14841–14846. 10.1073/pnas.1212454109 22927411 PMC3443146

[B53] Chehelgerdi M. Chehelgerdi M. Khorramian-Ghahfarokhi M. Shafieizadeh M. Mahmoudi E. Eskandari F. (2024). Comprehensive review of CRISPR-based gene editing: mechanisms, challenges, and applications in cancer therapy. Mol. Cancer 23, 9. 10.1186/s12943-023-01925-5 38195537 PMC10775503

[B54] Chen Y. Wan Y. Wang N. Yuan Z. Niu W. Li Q. (2018). Controlling the replication of a genomically recoded HIV-1 with a functional quadruplet codon in mammalian cells. ACS Synth. Biol. 7, 1612–1617. 10.1021/acssynbio.8b00096 29787233 PMC6003876

[B55] Chin J. W. (2014). Expanding and reprogramming the genetic code of cells and animals. Annu. Rev. Biochem. 83, 379–408. 10.1146/annurev-biochem-060713-035737 24555827

[B56] Chin J. W. (2017). Expanding and reprogramming the genetic code. Nature 550, 53–60. 10.1038/nature24031 28980641

[B57] Chua E. Y. D. Mendez J. H. Rapp M. Ilca S. L. Tan Y. Z. Maruthi K. (2022). Better, faster, cheaper: recent advances in cryo–electron microscopy. Annu. Rev. Biochem. 91, 1–32. 10.1146/annurev-biochem-032620-110705 35320683 PMC10393189

[B58] Chung C. Z. Krahn N. (2022). The selenocysteine toolbox: a guide to studying the 21st amino acid. Arch. Biochem. Biophys. 730, 109421. 10.1016/j.abb.2022.109421 36183842

[B59] Chung C. Z. Krahn N. Crnković A. Söll D. (2022). Intein-based design expands diversity of selenocysteine reporters. J. Mol. Biol. 434, 167199. 10.1016/j.jmb.2021.167199 34411545 PMC8847544

[B60] Cobb R. E. Chao R. Zhao H. (2013). Directed evolution: past, present and future. AIChE J. Am. Inst. Chem. Eng. 59, 1432–1440. 10.1002/aic.13995 PMC4344831 25733775

[B61] Coller J. Ignatova Z. (2024). tRNA therapeutics for genetic diseases. Nat. Rev. Drug Discov. 23, 108–125. 10.1038/s41573-023-00829-9 38049504

[B62] Crick F. H. C. (1966). Codon—anticodon pairing: the wobble hypothesis. J. Mol. Biol. 19, 548–555. 10.1016/S0022-2836(66)80022-0 5969078

[B63] Cruz-Vera L. R. Magos-Castro M. A. Zamora-Romo E. Guarneros G. (2004). Ribosome stalling and peptidyl-tRNA drop-off during translational delay at AGA codons. Nucleic Acids Res. 32, 4462–4468. 10.1093/nar/gkh784 15317870 PMC516057

[B64] Curran J. F. Yarus M. (1987). Reading frame selection and transfer RNA anticodon loop stacking. Science 238, 1545–1550. 10.1126/science.3685992 3685992

[B65] Dale T. Sanderson L. E. Uhlenbeck O. C. (2004). The affinity of elongation factor tu for an aminoacyl-tRNA is modulated by the esterified amino acid. Biochemistry 43, 6159–6166. 10.1021/bi036290o 15147200

[B66] Dale T. Uhlenbeck O. C. (2005). Amino acid specificity in translation. Trends biochem. Sci. 30, 659–665. 10.1016/j.tibs.2005.10.006 16260144

[B67] Davis L. Radman I. Goutou A. Tynan A. Baxter K. Xi Z. (2021). Precise optical control of gene expression in *C. elegans* using improved genetic code expansion and Cre recombinase. eLife 10, e67075. 10.7554/eLife.67075 34350826 PMC8448529

[B68] DeBenedictis E. A. Carver G. D. Chung C. Z. Söll D. Badran A. H. (2021). Multiplex suppression of four quadruplet codons via tRNA directed evolution. Nat. Commun. 12, 5706. 10.1038/s41467-021-25948-y 34588441 PMC8481270

[B69] DeBenedictis E. A. Söll D. Esvelt K. M. (2022). Measuring the tolerance of the genetic code to altered codon size. eLife 11, e76941. 10.7554/eLife.76941 35293861 PMC9094753

[B70] de la Torre D. Chin J. W. (2021). Reprogramming the genetic code. Nat. Rev. Genet. 22, 169–184. 10.1038/s41576-020-00307-7 33318706

[B71] Demeshkina N. Jenner L. Westhof E. Yusupov M. Yusupova G. (2012). A new understanding of the decoding principle on the ribosome. Nature 484, 256–259. 10.1038/nature10913 22437501

[B72] Dever T. E. Dinman J. D. Green R. (2018). Translation elongation and recoding in eukaryotes. Cold Spring Harb. Perspect. Biol. 10, a032649. 10.1101/cshperspect.a032649 29610120 PMC6071482

[B73] Djumagulov M. Demeshkina N. Jenner L. Rozov A. Yusupov M. Yusupova G. (2021). Accuracy mechanism of eukaryotic ribosome translocation. Nature 600, 543–546. 10.1038/s41586-021-04131-9 34853469 PMC8674143

[B74] Doerfel L. K. Wohlgemuth I. Kothe C. Peske F. Urlaub H. Rodnina M. V. (2013). EF-P is essential for rapid synthesis of proteins containing consecutive proline residues. Science 339, 85–88. 10.1126/science.1229017 23239624

[B75] Doerfel L. K. Wohlgemuth I. Kubyshkin V. Starosta A. L. Wilson D. N. Budisa N. (2015). Entropic contribution of elongation factor p to proline positioning at the catalytic center of the ribosome. J. Am. Chem. Soc. 137, 12997–13006. 10.1021/jacs.5b07427 26384033

[B76] Dolgin E. (2022). tRNA therapeutics burst onto startup scene. Nat. Biotechnol. 40, 283–286. 10.1038/s41587-022-01252-y 35210613

[B77] Dunkelmann D. L. Oehm S. B. Beattie A. T. Chin J. W. (2021). A 68-codon genetic code to incorporate four distinct non-canonical amino acids enabled by automated orthogonal mRNA design. Nat. Chem. 13, 1110–1117. 10.1038/s41557-021-00764-5 34426682 PMC7612796

[B78] Dunkelmann D. L. Willis J. C. W. Beattie A. T. Chin J. W. (2020). Engineered triply orthogonal pyrrolysyl–tRNA synthetase/tRNA pairs enable the genetic encoding of three distinct non-canonical amino acids. Nat. Chem. 12, 535–544. 10.1038/s41557-020-0472-x 32472101 PMC7116526

[B79] Eargle J. Black A. A. Sethi A. Trabuco L. G. Luthey-Schulten Z. (2008). Dynamics of recognition between tRNA and elongation factor tu. J. Mol. Biol. 377, 1382–1405. 10.1016/j.jmb.2008.01.073 18336835 PMC3232051

[B80] Elgamal S. Katz A. Hersch S. J. Newsom D. White P. Navarre W. W. (2014). EF-P dependent pauses integrate proximal and distal signals during translation. PLOS Genet. 10, e1004553. 10.1371/journal.pgen.1004553 25144653 PMC4140641

[B81] Ellefson J. W. Meyer A. J. Hughes R. A. Cannon J. R. Brodbelt J. S. Ellington A. D. (2014). Directed evolution of genetic parts and circuits by compartmentalized partnered replication. Nat. Biotechnol. 32, 97–101. 10.1038/nbt.2714 24185096

[B82] Englander M. T. Avins J. L. Fleisher R. C. Liu B. Effraim P. R. Wang J. (2015). The ribosome can discriminate the chirality of amino acids within its peptidyl-transferase center. Proc. Natl. Acad. Sci. 112, 6038–6043. 10.1073/pnas.1424712112 25918365 PMC4434717

[B83] Evans R. M. Krahn N. Murphy B. J. Lee H. Armstrong F. A. Söll D. (2021). Selective cysteine-to-selenocysteine changes in a [NiFe]-hydrogenase confirm a special position for catalysis and oxygen tolerance. Proc. Natl. Acad. Sci. U. S. A. 118, e2100921118. 10.1073/pnas.2100921118 33753519 PMC8020662

[B84] Evans R. M. Krahn N. Weiss J. Vincent K. A. Söll D. Armstrong F. A. (2024). Replacing a cysteine ligand by selenocysteine in a [NiFe]-hydrogenase unlocks hydrogen production activity and addresses the role of concerted proton-coupled electron transfer in electrocatalytic reversibility. J. Am. Chem. Soc. 10.1021/jacs.4c03489 PMC11212049 38747098

[B85] Fan C. Xiong H. Reynolds N. M. Söll D. (2015). Rationally evolving tRNA^Pyl^ for efficient incorporation of noncanonical amino acids. Nucleic Acids Res. 43, e156. 10.1093/nar/gkv800 26250114 PMC4678846

[B86] Fan Z. Song J. Guan T. Lv X. Wei J. (2018). Efficient expression of glutathione peroxidase with chimeric tRNA in amber-less *Escherichia coli* . ACS Synth. Biol. 7, 249–257. 10.1021/acssynbio.7b00290 28866886

[B87] Farabaugh P. J. (1996). Programmed translational frameshifting. Microbiol. Rev. 60, 103–134. 10.1128/mr.60.1.103-134.1996 8852897 PMC239420

[B88] Faulhammer H. G. Joshi R. L. (1987). Structural features in aminoacyl-tRNAs required for recognition by elongation factor tu. FEBS Lett. 217, 203–211. 10.1016/0014-5793(87)80664-6 3297780

[B89] Feng Z. Xu B. (2016). Inspiration from the mirror: d-amino acid containing peptides in biomedical approaches. Biomol. Concepts 7, 179–187. 10.1515/bmc-2015-0035 27159920 PMC5316480

[B90] Fischer N. Neumann P. Bock L. V. Maracci C. Wang Z. Paleskava A. (2016). The pathway to GTPase activation of elongation factor SelB on the ribosome. Nature 540, 80–85. 10.1038/nature20560 27842381

[B91] Fischer N. Neumann P. Konevega A. L. Bock L. V. Ficner R. Rodnina M. V. (2015). Structure of the *E. coli* ribosome–EF-Tu complex at <3 Å resolution by Cs-corrected cryo-EM. Nature 520, 567–570. 10.1038/nature14275 25707802

[B92] Fleisher R. C. Cornish V. W. Gonzalez R. L. Jr. (2018). d-amino acid-mediated translation arrest is modulated by the identity of the incoming aminoacyl-tRNA. Biochemistry 57, 4241–4246. 10.1021/acs.biochem.8b00595 29979035 PMC6330214

[B93] Frank J. Sengupta J. Gao H. Li W. Valle M. Zavialov A. (2005). The role of tRNA as a molecular spring in decoding, accommodation, and peptidyl transfer. FEBS Lett. 579, 959–962. 10.1016/j.febslet.2004.10.105 15680982

[B94] Fujino T. Goto Y. Suga H. Murakami H. (2013). Reevaluation of the d-amino acid compatibility with the elongation event in translation. J. Am. Chem. Soc. 135, 1830–1837. 10.1021/ja309570x 23301668

[B95] Ganesh R. B. Maerkl S. J. (2022). Biochemistry of aminoacyl tRNA synthetase and tRNAs and their engineering for cell-free and synthetic cell applications. Front. Bioeng. Biotechnol. 10, 918659. 10.3389/fbioe.2022.918659 35845409 PMC9283866

[B96] Gao Y.-G. Selmer M. Dunham C. M. Weixlbaumer A. Kelley A. C. Ramakrishnan V. (2009). The structure of the ribosome with elongation factor g trapped in the posttranslocational state. Science 326, 694–699. 10.1126/science.1179709 19833919 PMC3763468

[B97] Geslain R. Pan T. (2010). Functional analysis of human tRNA isodecoders. J. Mol. Biol. 396, 821–831. 10.1016/j.jmb.2009.12.018 20026070 PMC2822071

[B98] Giegé R. Eriani G. (2023). The tRNA identity landscape for aminoacylation and beyond. Nucleic Acids Res. 51, 1528–1570. 10.1093/nar/gkad007 36744444 PMC9976931

[B99] Giegé R. Frugier M. (2013). “Transfer RNA structure and identity,” in Madame curie bioscience database (Austin (TX): Landes Bioscience). Available at: https://www.ncbi.nlm.nih.gov/books/NBK6236/.

[B100] Giegé R. Jühling F. Pütz J. Stadler P. Sauter C. Florentz C. (2012). Structure of transfer RNAs: similarity and variability. WIREs RNA 3, 37–61. 10.1002/wrna.103 21957054

[B101] Gong X. Zhang H. Shen Y. Fu X. (2023). Update of the Pyrrolysyl-tRNA synthetase/tRNA^Pyl^ pair and derivatives for genetic code expansion. J. Bacteriol. 205, e0038522. 10.1128/jb.00385-22 36695595 PMC9945579

[B102] Gonzalez de Valdivia E. I. Isaksson L. A. (2005). Abortive translation caused by peptidyl-tRNA drop-off at NGG codons in the early coding region of mRNA. FEBS J. 272, 5306–5316. 10.1111/j.1742-4658.2005.04926.x 16218960

[B103] Goto Y. Murakami H. Suga H. (2008a). Initiating translation with D-amino acids. RNA 14, 1390–1398. 10.1261/rna.1020708 18515548 PMC2441986

[B104] Goto Y. Ohta A. Sako Y. Yamagishi Y. Murakami H. Suga H. (2008b). Reprogramming the translation initiation for the synthesis of physiologically stable cyclic peptides. ACS Chem. Biol. 3, 120–129. 10.1021/cb700233t 18215017

[B105] Goto Y. Suga H. (2021). The RaPID platform for the discovery of pseudo-natural macrocyclic peptides. Acc. Chem. Res. 54, 3604–3617. 10.1021/acs.accounts.1c00391 34505781

[B106] Gromadski K. B. Wieden H.-J. Rodnina M. V. (2002). Kinetic mechanism of elongation factor ts-catalyzed nucleotide exchange in elongation factor tu. Biochemistry 41, 162–169. 10.1021/bi015712w 11772013

[B107] Grosjean H. de Crécy-Lagard V. Marck C. (2010). Deciphering synonymous codons in the three domains of life: co-evolution with specific tRNA modification enzymes. FEBS Lett. 584, 252–264. 10.1016/j.febslet.2009.11.052 19931533

[B108] Grosjean H. Westhof E. (2016). An integrated, structure- and energy-based view of the genetic code. Nucleic Acids Res. 44, 8020–8040. 10.1093/nar/gkw608 27448410 PMC5041475

[B109] Guenneugues M. Caserta E. Brandi L. Spurio R. Meunier S. Pon C. L. (2000). Mapping the fMet‐tRNA^fMet^ binding site of initiation factor IF2. EMBO J. 19, 5233–5240. 10.1093/emboj/19.19.5233 11013225 PMC302095

[B110] Guo J. Melançon C. E. Lee H. S. Groff D. Schultz P. G. (2009). Evolution of amber suppressor tRNAs for efficient bacterial production of proteins containing nonnatural amino acids. Angew. Chem. Int. Ed. Engl. 48, 9148–9151. 10.1002/anie.200904035 19856359 PMC2887739

[B111] Guo J. Niu W. (2022). Genetic code expansion through quadruplet codon decoding. J. Mol. Biol. 434, 167346. 10.1016/j.jmb.2021.167346 34762896 PMC9018476

[B112] Gutierrez E. Shin B.-S. Woolstenhulme C. J. Kim J.-R. Saini P. Buskirk A. R. (2013). eIF5A promotes translation of polyproline motifs. Mol. Cell 51, 35–45. 10.1016/j.molcel.2013.04.021 23727016 PMC3744875

[B113] Hammerling M. J. Krüger A. Jewett M. C. (2020). Strategies for *in vitro* engineering of the translation machinery. Nucleic Acids Res. 48, 1068–1083. 10.1093/nar/gkz1011 31777928 PMC7026604

[B114] Hankore E. D. Zhang L. Chen Y. Liu K. Niu W. Guo J. (2019). Genetic incorporation of noncanonical amino acids using two mutually orthogonal quadruplet codons. ACS Synth. Biol. 8, 1168–1174. 10.1021/acssynbio.9b00051 30995842 PMC6525065

[B115] Hoffman K. S. Chung C. Z. Mukai T. Krahn N. Jiang H.-K. Balasuriya N. (2023). Recoding UAG to selenocysteine in *Saccharomyces cerevisiae* . RNA 29, 1400–1410. 10.1261/rna.079658.123 37279998 PMC10573291

[B116] Hohsaka T. Ashizuka Y. Murakami H. Sisido M. (1996). Incorporation of nonnatural amino acids into streptavidin through *in vitro* frame-shift suppression. J. Am. Chem. Soc. 118, 9778–9779. 10.1021/ja9614225

[B117] Hohsaka T. Ashizuka Y. Sasaki H. Murakami H. Sisido M. (1999). Incorporation of two different nonnatural amino acids independently into a single protein through extension of the genetic code. J. Am. Chem. Soc. 121, 12194–12195. 10.1021/ja992204p

[B118] Hohsaka T. Ashizuka Y. Taira H. Murakami H. Sisido M. (2001). Incorporation of nonnatural amino acids into proteins by using various four-base codons in an *Escherichia coli in vitro* translation Ssystem. Biochemistry 40, 11060–11064. 10.1021/bi0108204 11551202

[B119] Holley R. W. Apgar J. Everett G. A. Madison J. T. Marquisee M. Merrill S. H. (1965). Structure of a ribonucleic acid. Science 147, 1462–1465. 10.1126/science.147.3664.1462 14263761

[B120] Hou Y. Zhang W. McGilvray P. T. Sobczyk M. Wang T. Weng S. H. S. (2024). Engineered mischarged transfer RNAs for correcting pathogenic missense mutations. Mol. Ther. 32, 352–371. 10.1016/j.ymthe.2023.12.014 38104240 PMC10861979

[B121] Huang R. L. Jewel D. Kelemen R. E. Pham Q. Wang S. Roy S. J. S. (2024). Directed evolution of a bacterial leucyl tRNA in mammalian cells for enhanced noncanonical amino acid mutagenesis. bioRxiv. 2024.02.19.581038. 10.1101/2024.02.19.581038 PMC11789822 38904157

[B122] Hummels K. R. Kearns D. B. (2020). Translation elongation factor p (EF-P). FEMS Microbiol. Rev. 44, 208–218. 10.1093/femsre/fuaa003 32011712 PMC7269679

[B123] Hussain T. Llácer J. L. Wimberly B. T. Kieft J. S. Ramakrishnan V. (2016). Large-scale movements of IF3 and tRNA during bacterial translation initiation. Cell 167, 133–144. 10.1016/j.cell.2016.08.074 27662086 PMC5037330

[B124] Huter P. Arenz S. Bock L. V. Graf M. Frister J. O. Heuer A. (2017). Structural basis for polyproline-mediated ribosome stalling and rescue by the translation elongation factor EF-P. Mol. Cell 68, 515–527. 10.1016/j.molcel.2017.10.014 29100052

[B125] Ieong K.-W. Pavlov M. Y. Kwiatkowski M. Ehrenberg M. Forster A. C. (2014). A tRNA body with high affinity for EF-Tu hastens ribosomal incorporation of unnatural amino acids. RNA 20, 632–643. 10.1261/rna.042234.113 24671767 PMC3988565

[B126] Italia J. S. Zheng Y. Kelemen R. E. Erickson S. B. Addy P. S. Chatterjee A. (2017). Expanding the genetic code of mammalian cells. Biochem. Soc. Trans. 45, 555–562. 10.1042/BST20160336 28408495

[B127] Itoh Y. Bröcker M. J. Sekine S. Hammond G. Suetsugu S. Söll D. (2013a). Decameric SelA·tRNA^Sec^ ring structure reveals mechanism of bacterial selenocysteine formation. Science 340, 75–78. 10.1126/science.1229521 23559248 PMC3976565

[B128] Itoh Y. Sekine S. Suetsugu S. Yokoyama S. (2013b). Tertiary structure of bacterial selenocysteine tRNA. Nucleic Acids Res. 41, 6729–6738. 10.1093/nar/gkt321 23649835 PMC3711452

[B129] Jewel D. Kelemen R. E. Huang R. L. Zhu Z. Sundaresh B. Cao X. (2023). Virus-assisted directed evolution of enhanced suppressor tRNAs in mammalian cells. Nat. Methods 20, 95–103. 10.1038/s41592-022-01706-w 36550276 PMC9855281

[B130] Jewel D. Kelemen R. E. Huang R. L. Zhu Z. Sundaresh B. Malley K. (2024). Enhanced directed evolution in mammalian cells yields a hyperefficient pyrrolysyl tRNA for noncanonical amino acid mutagenesis. Angew. Chem. Int. Ed. 63, e202316428. 10.1002/anie.202316428 PMC10922736 38279536

[B131] Jobe A. Liu Z. Gutierrez-Vargas C. Frank J. (2019). New insights into ribosome structure and function. Cold Spring Harb. Perspect. Biol. 11, a032615. 10.1101/cshperspect.a032615 29903714 PMC6314068

[B132] Johansen J. S. Kavaliauskas D. Pfeil S. H. Blaise M. Cooperman B. S. Goldman Y. E. (2018). *E. coli* elongation factor tu bound to a GTP analogue displays an open conformation equivalent to the GDP-bound form. Nucleic Acids Res. 46, 8641–8650. 10.1093/nar/gky697 30107565 PMC6144822

[B133] Jühling F. Mörl M. Hartmann R. K. Sprinzl M. Stadler P. F. Pütz J. (2009). tRNAdb 2009: compilation of tRNA sequences and tRNA genes. Nucleic Acids Res. 37, D159–D162. 10.1093/nar/gkn772 18957446 PMC2686557

[B134] Kaiser F. Krautwurst S. Salentin S. Haupt V. J. Leberecht C. Bittrich S. (2020). The structural basis of the genetic code: amino acid recognition by aminoacyl-tRNA synthetases. Sci. Rep. 10, 12647. 10.1038/s41598-020-69100-0 32724042 PMC7387524

[B135] Kaledhonkar S. Fu Z. Caban K. Li W. Chen B. Sun M. (2019). Late steps in bacterial translation initiation visualized using time-resolved cryo-EM. Nature 570, 400–404. 10.1038/s41586-019-1249-5 31108498 PMC7060745

[B136] Kang T. J. Suga H. (2011). Translation of a histone H3 tail as a model system for studying peptidyl-tRNA drop-off. FEBS Lett. 585, 2269–2274. 10.1016/j.febslet.2011.05.051 21627973

[B137] Katoh T. Iwane Y. Suga H. (2017a). Logical engineering of D-arm and T-stem of tRNA that enhances d-amino acid incorporation. Nucleic Acids Res. 45, 12601–12610. 10.1093/nar/gkx1129 29155943 PMC5728406

[B138] Katoh T. Sengoku T. Hirata K. Ogata K. Suga H. (2020). Ribosomal synthesis and *de novo* discovery of bioactive foldamer peptides containing cyclic β-amino acids. Nat. Chem. 12, 1081–1088. 10.1038/s41557-020-0525-1 32839601

[B139] Katoh T. Suga H. (2018). Ribosomal incorporation of consecutive β-amino acids. J. Am. Chem. Soc. 140, 12159–12167. 10.1021/jacs.8b07247 30221942

[B140] Katoh T. Suga H. (2022a). *In vitro* genetic code reprogramming for the expansion of useable noncanonical amino acids. Annu. Rev. Biochem. 91, 221–243. 10.1146/annurev-biochem-040320-103817 35729073

[B141] Katoh T. Suga H. (2022b). *In vitro* selection of foldamer-like macrocyclic peptides containing 2-aminobenzoic acid and 3-aminothiophene-2-carboxylic acid. J. Am. Chem. Soc. 144, 2069–2072. 10.1021/jacs.1c12133 35099961

[B142] Katoh T. Suga H. (2023a). Drop-off-reinitiation at the amino termini of nascent peptides and its regulation by IF3, EF-G, and RRF. RNA 29, 663–674. 10.1261/rna.079447.122 36754577 PMC10158994

[B143] Katoh T. Suga H. (2023b). Ribosomal incorporation of negatively charged d-α- and N-methyl-l-α-amino acids enhanced by EF-Sep. Philos. Trans. R. Soc. B Biol. Sci. 378, 20220038. 10.1098/rstb.2022.0038 PMC9835608 36633283

[B144] Katoh T. Suga H. (2023c). Translation initiation with exotic amino acids using EF-P-responsive artificial initiator tRNA. Nucleic Acids Res. 51, 8169–8180. 10.1093/nar/gkad496 37334856 PMC10450175

[B145] Katoh T. Suga H. (2024). Fine-tuning the tRNA anticodon arm for multiple/consecutive incorporations of β-amino acids and analogs. Nucleic Acids Res., gkae219. 10.1093/nar/gkae219 PMC11194099 38572748

[B146] Katoh T. Tajima K. Suga H. (2017b). Consecutive elongation of d-amino acids in translation. Cell Chem. Biol. 24, 46–54. 10.1016/j.chembiol.2016.11.012 28042044

[B147] Katoh T. Wohlgemuth I. Nagano M. Rodnina M. V. Suga H. (2016). Essential structural elements in tRNA^Pro^ for EF-P-mediated alleviation of translation stalling. Nat. Commun. 7, 11657. 10.1038/ncomms11657 27216360 PMC4890201

[B148] Katsonis P. Koire A. Wilson S. J. Hsu T.-K. Lua R. C. Wilkins A. D. (2014). Single nucleotide variations: biological impact and theoretical interpretation. Protein Sci. 23, 1650–1666. 10.1002/pro.2552 25234433 PMC4253807

[B149] Kaul G. Pattan G. Rafeequi T. (2011). Eukaryotic elongation factor-2 (eEF2): its regulation and peptide chain elongation. Cell biochem. Funct. 29, 227–234. 10.1002/cbf.1740 21394738

[B150] Kavaliauskas D. Nissen P. Knudsen C. R. (2012). The busiest of all ribosomal assistants: elongation factor tu. Biochemistry 51, 2642–2651. 10.1021/bi300077s 22409271

[B151] Kawashima T. Berthet-Colominas C. Wulff M. Cusack S. Leberman R. (1996). The structure of the *Escherichia coli* EF-Tu.EF-Ts complex at 2.5 Å resolution. Nature 379, 511–518. 10.1038/379511a0 8596629

[B152] Kim S. H. Sussman J. L. Suddath F. L. Quigley G. J. McPherson A. Wang A. H. J. (1974). The general structure of transfer RNA molecules. Proc. Natl. Acad. Sci. 71, 4970–4974. 10.1073/pnas.71.12.4970 4612535 PMC434021

[B153] Kim Y. Cho S. Kim J.-C. Park H.-S. (2024). tRNA engineering strategies for genetic code expansion. Front. Genet. 15, 1373250. 10.3389/fgene.2024.1373250 38516376 PMC10954879

[B154] Kjeldgaard M. Nissen P. Thirup S. Nyborg J. (1993). The crystal structure of elongation factor EF-Tu from *Thermus aquaticus* in the GTP conformation. Structure 1, 35–50. 10.1016/0969-2126(93)90007-4 8069622

[B155] Kleina L. G. Masson J.-M. Normanly J. Abelson J. Miller J. H. (1990). Construction of *Escherichia coli* amber suppressor tRNA genes. J. Mol. Biol. 213, 705–717. 10.1016/S0022-2836(05)80257-8 2193162

[B156] Ko W. Porter J. J. Sipple M. T. Edwards K. M. Lueck J. D. (2022). Efficient suppression of endogenous CFTR nonsense mutations using anticodon-engineered transfer RNAs. Mol. Ther. - Nucleic Acids 28, 685–701. 10.1016/j.omtn.2022.04.033 35664697 PMC9126842

[B157] Komoda T. Sato N. S. Phelps S. S. Namba N. Joseph S. Suzuki T. (2006). The A-site finger in 23 S rRNA acts as a functional attenuator for translocation. J. Biol. Chem. 281, 32303–32309. 10.1074/jbc.M607058200 16950778

[B158] Korostelev A. A. (2022). The structural dynamics of translation. Annu. Rev. Biochem. 91, 245–267. 10.1146/annurev-biochem-071921-122857 35287473 PMC10389292

[B159] Krahn N. Fischer J. T. Söll D. (2020a). Naturally occurring tRNAs with non-canonical structures. Front. Microbiol. 11, 596914. 10.3389/fmicb.2020.596914 33193279 PMC7609411

[B160] Krahn N. Tharp J. M. Crnković A. Söll D. (2020b). Engineering aminoacyl-tRNA synthetases for use in synthetic biology. Enzym. 48, 351–395. 10.1016/bs.enz.2020.06.004 PMC8086897 33837709

[B161] Krahn N. Zhang J. Melnikov S. V. Tharp J. M. Villa A. Patel A. (2024). tRNA shape is an identity element for an archaeal pyrrolysyl-tRNA synthetase from the human gut. Nucleic Acids Res. 52, 513–524. 10.1093/nar/gkad1188 38100361 PMC10810272

[B162] Kumbhar B. V. Kamble A. D. Sonawane K. D. (2013). Conformational preferences of modified nucleoside N(4)-acetylcytidine, ac4C occur at “wobble” 34th position in the anticodon loop of tRNA. Cell biochem. Biophys. 66, 797–816. 10.1007/s12013-013-9525-8 23408308

[B163] Kurata S. Weixlbaumer A. Ohtsuki T. Shimazaki T. Wada T. Kirino Y. (2008). Modified uridines with C5-methylene substituents at the first position of the tRNA anticodon stabilize U.G wobble pairing during decoding. J. Biol. Chem. 283, 18801–18811. 10.1074/jbc.M800233200 18456657

[B164] Lajoie M. J. Rovner A. J. Goodman D. B. Aerni H.-R. Haimovich A. D. Kuznetsov G. (2013). Genomically recoded organisms expand biological functions. Science 342, 357–360. 10.1126/science.1241459 24136966 PMC4924538

[B165] Lapointe C. P. Grosely R. Sokabe M. Alvarado C. Wang J. Montabana E. (2022). eIF5B and eIF1A reorient initiator tRNA to allow ribosomal subunit joining. Nature 607, 185–190. 10.1038/s41586-022-04858-z 35732735 PMC9728550

[B166] LaRiviere F. J. Wolfson A. D. Uhlenbeck O. C. (2001). Uniform binding of aminoacyl-tRNAs to elongation factor tu by thermodynamic compensation. Science 294, 165–168. 10.1126/science.1064242 11588263

[B167] Ledoux S. Olejniczak M. Uhlenbeck O. C. (2009). A sequence element that tunes *Escherichia coli* tRNA^Ala^ _GGC_ to ensure accurate decoding. Nat. Struct. Mol. Biol. 16, 359–364. 10.1038/nsmb.1581 19305403 PMC2769084

[B168] Ledoux S. Uhlenbeck O. C. (2008). Different aa-tRNAs are selected uniformly on the ribosome. Mol. Cell 31, 114–123. 10.1016/j.molcel.2008.04.026 18614050 PMC2709977

[B169] Lee B. S. Choi W. J. Lee S. W. Ko B. J. Yoo T. H. (2021a). Towards engineering an orthogonal protein translation initiation system. Front. Chem. 9, 772648. 10.3389/fchem.2021.772648 34765589 PMC8576571

[B170] Lee J. Coronado J. N. Cho N. Lim J. Hosford B. M. Seo S. (2022). Ribosome-mediated biosynthesis of pyridazinone oligomers *in vitro* . Nat. Commun. 13, 6322. 10.1038/s41467-022-33701-2 36280685 PMC9592601

[B171] Lee J. Schwarz K. J. Kim D. S. Moore J. S. Jewett M. C. (2020a). Ribosome-mediated polymerization of long chain carbon and cyclic amino acids into peptides *in vitro* . Nat. Commun. 11, 4304. 10.1038/s41467-020-18001-x 32855412 PMC7452890

[B172] Lee J. Schwarz K. J. Yu H. Krüger A. Anslyn E. V. Ellington A. D. (2021b). Ribosome-mediated incorporation of fluorescent amino acids into peptides *in vitro* . Chem. Commun. 57, 2661–2664. 10.1039/D0CC07740B 33592078

[B173] Lee J. Torres R. Kim D. S. Byrom M. Ellington A. D. Jewett M. C. (2020b). Ribosomal incorporation of cyclic β-amino acids into peptides using *in vitro* translation. Chem. Commun. 56, 5597–5600. 10.1039/D0CC02121K 32400780

[B174] Liljeruhm J. Wang J. Kwiatkowski M. Sabari S. Forster A. C. (2019). Kinetics of d-amino acid incorporation in translation. ACS Chem. Biol. 14, 204–213. 10.1021/acschembio.8b00952 30648860

[B175] Lin T.-Y. Glatt S. (2022). ACEing premature codon termination using anticodon-engineered sup-tRNA-based therapy. Mol. Ther. Nucleic Acids 29, 368–369. 10.1016/j.omtn.2022.07.019 36035751 PMC9386023

[B176] Ling C. Ermolenko D. N. (2016). Structural insights into ribosome translocation. Wiley Interdiscip. Rev. RNA 7, 620–636. 10.1002/wrna.1354 27117863 PMC4990484

[B177] Liu C.-Y. Qureshi M. T. Lee T.-H. (2011). Interaction strengths between the ribosome and tRNA at various steps of translocation. Biophys. J. 100, 2201–2208. 10.1016/j.bpj.2011.03.023 21539788 PMC3149268

[B178] Liu G. Song G. Zhang D. Zhang D. Li Z. Lyu Z. (2014). EF-G catalyzes tRNA translocation by disrupting interactions between decoding center and codon–anticodon duplex. Nat. Struct. Mol. Biol. 21, 817–824. 10.1038/nsmb.2869 25108354

[B179] Lorenz C. Lünse C. E. Mörl M. (2017). tRNA modifications: impact on structure and thermal adaptation. Biomolecules 7, 35. 10.3390/biom7020035 28375166 PMC5485724

[B180] Louie A. Jurnak F. (1985). Kinetic studies of *Escherichia coli* elongation factor tu-guanosine 5’-triphosphate-aminoacyl-tRNA complexes. Biochemistry 24, 6433–6439. 10.1021/bi00344a019 3910093

[B181] Louie A. Ribeiro N. S. Reid B. R. Jurnak F. (1984). Relative affinities of all *Escherichia coli* aminoacyl-tRNAs for elongation factor tu-GTP. J. Biol. Chem. 259, 5010–5016. 10.1016/s0021-9258(17)42947-4 6370998

[B182] Loveland A. B. Demo G. Grigorieff N. Korostelev A. A. (2017). Ensemble cryo-EM elucidates the mechanism of translation fidelity. Nature 546, 113–117. 10.1038/nature22397 28538735 PMC5657493

[B183] Lowe T. M. Chan P. P. (2016). tRNAscan-SE on-line: integrating search and context for analysis of transfer RNA genes. Nucleic Acids Res. 44, W54–W57. 10.1093/nar/gkw413 27174935 PMC4987944

[B184] Lueck J. D. Yoon J. S. Perales-Puchalt A. Mackey A. L. Infield D. T. Behlke M. A. (2019). Engineered transfer RNAs for suppression of premature termination codons. Nat. Commun. 10, 822. 10.1038/s41467-019-08329-4 30778053 PMC6379413

[B185] Ma C. Kudlicki W. Odom O. W. Kramer G. Hardesty B. (1993). *In vitro* protein engineering using synthetic tRNA^Ala^ with different anticodons. Biochemistry 32, 7939–7945. 10.1021/bi00082a015 8347599

[B186] Machnicka M. A. Olchowik A. Grosjean H. Bujnicki J. M. (2014). Distribution and frequencies of post-transcriptional modifications in tRNAs. RNA Biol. 11, 1619–1629. 10.4161/15476286.2014.992273 25611331 PMC4615829

[B187] Maehigashi T. Dunkle J. A. Miles S. J. Dunham C. M. (2014). Structural insights into +1 frameshifting promoted by expanded or modification-deficient anticodon stem loops. Proc. Natl. Acad. Sci. 111, 12740–12745. 10.1073/pnas.1409436111 25128388 PMC4156745

[B188] Magliery T. J. Anderson J. C. Schultz P. G. (2001). Expanding the genetic code: selection of efficient suppressors of four-base codons and identification of “shifty” four-base codons with a library approach in *Escherichia coli* . J. Mol. Biol. 307, 755–769. 10.1006/jmbi.2001.4518 11273699 PMC7125544

[B189] Maglott E. J. Goodwin J. T. Glick G. D. (1999). Probing the structure of an RNA tertiary unfolding transition state. J. Am. Chem. Soc. 121, 7461–7462. 10.1021/ja9913075

[B190] Mala P. Saraogi I. (2022). Enhanced codon–anticodon interaction at in-frame UAG stop codon improves the efficiency of non-natural amino acid mutagenesis. ACS Chem. Biol. 17, 1051–1060. 10.1021/acschembio.1c00782 35532803

[B191] Mandal N. Mangroo D. Dalluge J. J. McCloskey J. A. Rajbhandary U. L. (1996). Role of the three consecutive G:C base pairs conserved in the anticodon stem of initiator tRNAs in initiation of protein synthesis in *Escherichia coli* . RNA 2, 473–482. 8665414 PMC1369388

[B192] Maranhao A. C. Ellington A. D. (2017). Evolving orthogonal suppressor tRNAs to incorporate modified amino acids. ACS Synth. Biol. 6, 108–119. 10.1021/acssynbio.6b00145 27600875

[B193] Mariotti M. Salinas G. Gabaldón T. Gladyshev V. N. (2019). Utilization of selenocysteine in early-branching fungal phyla. Nat. Microbiol. 4, 759–765. 10.1038/s41564-018-0354-9 30742068 PMC6551623

[B194] Matassi G. (2017). Horizontal gene transfer drives the evolution of Rh50 permeases in prokaryotes. BMC Evol. Biol. 17, 2. 10.1186/s12862-016-0850-6 28049420 PMC5209957

[B195] McFeely C. A. L. Dods K. K. Patel S. S. Hartman M. C. T. (2022). Expansion of the genetic code through reassignment of redundant sense codons using fully modified tRNA. Nucleic Acids Res. 50, 11374–11386. 10.1093/nar/gkac846 36300637 PMC9638912

[B196] McIntosh J. A. Donia M. S. Schmidt E. W. (2009). Ribosomal peptide natural products: bridging the ribosomal and nonribosomal worlds. Nat. Prod. Rep. 26, 537–559. 10.1039/b714132g 19642421 PMC2975598

[B197] Melchionna M. Styan K. E. Marchesan S. (2016). The unexpected advantages of using d-amino acids for peptide self-assembly into nanostructured hydrogels for medicine. Curr. Top. Med. Chem. 16, 2009–2018. 10.2174/1568026616999160212120302 26876522 PMC5374841

[B198] Melnikov S. Mailliot J. Rigger L. Neuner S. Shin B. Yusupova G. (2016a). Molecular insights into protein synthesis with proline residues. EMBO Rep. 17, 1776–1784. 10.15252/embr.201642943 27827794 PMC5283605

[B199] Melnikov S. Mailliot J. Shin B.-S. Rigger L. Yusupova G. Micura R. (2016b). Crystal structure of hypusine-containing translation factor eIF5A bound to a rotated eukaryotic ribosome. J. Mol. Biol. 428, 3570–3576. 10.1016/j.jmb.2016.05.011 27196944 PMC5408928

[B200] Melnikov S. V. Khabibullina N. F. Mairhofer E. Vargas-Rodriguez O. Reynolds N. M. Micura R. (2019). Mechanistic insights into the slow peptide bond formation with D-amino acids in the ribosomal active site. Nucleic Acids Res. 47, 2089–2100. 10.1093/nar/gky1211 30520988 PMC6393236

[B201] Melnikov S. V. Söll D. (2019). Aminoacyl-tRNA synthetases and tRNAs for an expanded genetic code: what makes them orthogonal? Int. J. Mol. Sci. 20, 1929. 10.3390/ijms20081929 31010123 PMC6515474

[B202] Menninger J. R. (1976). Peptidyl transfer RNA dissociates during protein synthesis from ribosomes of *Escherichia coli* . J. Biol. Chem. 251, 3392–3398. 10.1016/S0021-9258(17)33450-6 776968

[B203] Milicevic N. Jenner L. Myasnikov A. Yusupov M. Yusupova G. (2024). mRNA reading frame maintenance during eukaryotic ribosome translocation. Nature 625, 393–400. 10.1038/s41586-023-06780-4 38030725

[B204] Miller C. Bröcker M. J. Prat L. Ip K. Chirathivat N. Feiock A. (2015). A synthetic tRNA for EF-Tu mediated selenocysteine incorporation *in vivo* and *in vitro* . FEBS Lett. 589, 2194–2199. 10.1016/j.febslet.2015.06.039 26160755 PMC4782793

[B205] Miller D. L. Weissbach H. (1970). Interactions between the elongation factors: the displacement of GPD from the TU-GDP complex by factor Ts. Biochem. Biophys. Res. Commun. 38, 1016–1022. 10.1016/0006-291X(70)90341-4 5432207

[B206] Miller S. M. Wang T. Liu D. R. (2020). Phage-assisted continuous and non-continuous evolution. Nat. Protoc. 15, 4101–4127. 10.1038/s41596-020-00410-3 33199872 PMC7865204

[B207] Mills E. M. Barlow V. L. Jones A. T. Tsai Y.-H. (2021). Development of mammalian cell logic gates controlled by unnatural amino acids. Cell Rep. Methods 1, 100073. 10.1016/j.crmeth.2021.100073 35474893 PMC9017196

[B208] Milon P. Carotti M. Konevega A. L. Wintermeyer W. Rodnina M. V. Gualerzi C. O. (2010). The ribosome‐bound initiation factor 2 recruits initiator tRNA to the 30S initiation complex. EMBO Rep. 11, 312–316. 10.1038/embor.2010.12 20224578 PMC2854590

[B209] Milón P. Rodnina M. V. (2012). Kinetic control of translation initiation in bacteria. Crit. Rev. Biochem. Mol. Biol. 47, 334–348. 10.3109/10409238.2012.678284 22515367

[B210] Miyajima A. Kaziro Y. (1978). Coordination of levels of elongation factors tu, ts, and g, and ribosomal protein SI in *Escherichia coli* . J. Biochem. (Tokyo) 83, 453–462. 10.1093/oxfordjournals.jbchem.a131932 344309

[B211] Mohanta T. K. Mohanta Y. K. Sharma N. (2023). Anticodon table of the chloroplast genome and identification of putative quadruplet anticodons in chloroplast tRNAs. Sci. Rep. 13, 760. 10.1038/s41598-023-27886-9 36641535 PMC9840617

[B212] Moore B. Nelson C. C. Persson B. C. Gesteland R. F. Atkins J. F. (2000a). Decoding of tandem quadruplets by adjacent tRNAs with eight-base anticodon loops. Nucleic Acids Res. 28, 3615–3624. 10.1093/nar/28.18.3615 10982884 PMC110719

[B213] Moore B. Persson B. C. Nelson C. C. Gesteland R. F. Atkins J. F. (2000b). Quadruplet codons: implications for code expansion and the specification of translation step size. J. Mol. Biol. 298, 195–209. 10.1006/jmbi.2000.3658 10764591

[B214] Morosky P. Comyns C. Nunes L. G. A. Chung C. Z. Hoffmann P. R. Söll D. (2023). Dual incorporation of non-canonical amino acids enables production of post-translationally modified selenoproteins. Front. Mol. Biosci. 10, 1096261. 10.3389/fmolb.2023.1096261 36762212 PMC9902344

[B215] Mort M. Ivanov D. Cooper D. N. Chuzhanova N. A. (2008). A meta-analysis of nonsense mutations causing human genetic disease. Hum. Mutat. 29, 1037–1047. 10.1002/humu.20763 18454449

[B216] Mousa R. Notis Dardashti R. Metanis N. (2017). Selenium and selenocysteine in protein chemistry. Angew. Chem. Int. Ed. 56, 15818–15827. 10.1002/anie.201706876 28857389

[B217] Mukai T. Englert M. Tripp H. J. Miller C. Ivanova N. N. Rubin E. M. (2016). Facile recoding of selenocysteine in nature. Angew. Chem. Int. Ed. Engl. 55, 5337–5341. 10.1002/anie.201511657 26991476 PMC4833512

[B218] Mukai T. Sevostyanova A. Suzuki T. Fu X. Söll D. (2018). A facile method for producing selenocysteine-containing proteins. Angew. Chem. Int. Ed. Engl. 57, 7215–7219. 10.1002/anie.201713215 29631320 PMC6035045

[B219] Mukai T. Vargas-Rodriguez O. Englert M. Tripp H. J. Ivanova N. N. Rubin E. M. (2017). Transfer RNAs with novel cloverleaf structures. Nucleic Acids Res. 45, 2776–2785. 10.1093/nar/gkw898 28076288 PMC5389517

[B220] Murakami H. Ohta A. Suga H. (2009). Bases in the anticodon loop of tRNA^Ala^ _GGC_ prevent misreading. Nat. Struct. Mol. Biol. 16, 353–358. 10.1038/nsmb.1580 19305404

[B221] Muto H. Ito K. (2008). Peptidyl-prolyl-tRNA at the ribosomal P-site reacts poorly with puromycin. Biochem. Biophys. Res. Commun. 366, 1043–1047. 10.1016/j.bbrc.2007.12.072 18155161

[B222] Nakano S. Suzuki T. Kawarada L. Iwata H. Asano K. Suzuki T. (2016). NSUN3 methylase initiates 5-formylcytidine biogenesis in human mitochondrial tRNA^Met^ . Nat. Chem. Biol. 12, 546–551. 10.1038/nchembio.2099 27214402

[B223] Nedialkova D. D. Leidel S. A. (2015). Optimization of codon translation rates via tRNA modifications maintains proteome integrity. Cell 161, 1606–1618. 10.1016/j.cell.2015.05.022 26052047 PMC4503807

[B224] Neelagandan N. Lamberti I. Carvalho H. J. F. Gobet C. Naef F. (2020). What determines eukaryotic translation elongation: recent molecular and quantitative analyses of protein synthesis. Open Biol. 10, 200292. 10.1098/rsob.200292 33292102 PMC7776565

[B225] Neumann H. Wang K. Davis L. Garcia-Alai M. Chin J. W. (2010). Encoding multiple unnatural amino acids via evolution of a quadruplet-decoding ribosome. Nature 464, 441–444. 10.1038/nature08817 20154731

[B226] Nguyen H. A. Sunita S. Dunham C. M. (2020). Disruption of evolutionarily correlated tRNA elements impairs accurate decoding. Proc. Natl. Acad. Sci. 117, 16333–16338. 10.1073/pnas.2004170117 32601241 PMC7368331

[B227] Nguyen K. Whitford P. C. (2016). Capturing transition states for tRNA hybrid-state formation in the ribosome. J. Phys. Chem. B 120, 8768–8775. 10.1021/acs.jpcb.6b04476 27479146

[B228] Nguyen K. Yang H. Whitford P. C. (2017). How the ribosomal a-site finger can lead to tRNA species-dependent dynamics. J. Phys. Chem. B 121, 2767–2775. 10.1021/acs.jpcb.7b01072 28276690

[B229] Nishimuka S. (1972). “Minor components in transfer RNA: their characterization, location, and function,” in Progress in nucleic acid research and molecular biology. Editors Davidson, J. N. Cohn W. E. (Academic Press), 49–85. 10.1016/S0079-6603(08)60659-5 4557059

[B230] Nissen P. Kjeldgaard M. Thirup S. Clark B. F. C. Nyborg J. (1996). The ternary complex of aminoacylated tRNA and EF-Tu-GTP. Recognition of a bond and a fold. Biochimie 78, 921–933. 10.1016/S0300-9084(97)86714-4 9150869

[B231] Nissen P. Kjeldgaard M. Thirup S. Polekhina G. Reshetnikova L. Clark B. F. C. (1995). Crystal structure of the ternary complex of Phe-tRNA^Phe^, EF-Tu, and a GTP analog. Science 270, 1464–1472. 10.1126/science.270.5241.1464 7491491

[B232] Nissen P. Thirup S. Kjeldgaard M. Nyborg J. (1999). The crystal structure of Cys-tRNACys–EF-Tu–GDPNP reveals general and specific features in the ternary complex and in tRNA. Structure 7, 143–156. 10.1016/S0969-2126(99)80021-5 10368282

[B233] Niu W. Schultz P. G. Guo J. (2013). An expanded genetic code in mammalian cells with a functional quadruplet codon. ACS Chem. Biol. 8, 1640–1645. 10.1021/cb4001662 23662731 PMC4501474

[B234] Nozawa K. O’Donoghue P. Gundllapalli S. Araiso Y. Ishitani R. Umehara T. (2009). Pyrrolysyl-tRNA synthetase-tRNA^Pyl^ structure reveals the molecular basis of orthogonality. Nature 457, 1163–1167. 10.1038/nature07611 19118381 PMC2648862

[B235] O’Connor M. (2002). Insertions in the anticodon loop of tRNA_1_ ^Gln^ (sufG) and tRNA^Lys^ promote quadruplet decoding of CAAA. Nucleic Acids Res. 30, 1985–1990. 10.1093/nar/30.9.1985 11972336 PMC113831

[B236] Ogawa A. Doi Y. Matsushita N. (2011). Improvement of *in vitro*-transcribed amber suppressor tRNAs toward higher suppression efficiency in wheat germ extract. Org. Biomol. Chem. 9, 8495–8503. 10.1039/C1OB06351K 22068346

[B237] Ogawa A. Hayami M. Sando S. Aoyama Y. (2012). A concept for selection of codon-suppressor tRNAs based on read-through ribosome display in an *in vitro* compartmentalized cell-free translation system. J. Nucleic Acids 2012, 538129. 10.1155/2012/538129 22928090 PMC3425794

[B238] Ohtsuki T. Manabe T. Sisido M. (2005). Multiple incorporation of non-natural amino acids into a single protein using tRNAs with non-standard structures. FEBS Lett. 579, 6769–6774. 10.1016/j.febslet.2005.11.010 16310775

[B239] Olejniczak M. Dale T. Fahlman R. P. Uhlenbeck O. C. (2005). Idiosyncratic tuning of tRNAs to achieve uniform ribosome binding. Nat. Struct. Mol. Biol. 12, 788–793. 10.1038/nsmb978 16116437

[B240] Olejniczak M. Uhlenbeck O. C. (2006). tRNA residues that have coevolved with their anticodon to ensure uniform and accurate codon recognition. Biochimie 88, 943–950. 10.1016/j.biochi.2006.06.005 16828219

[B241] Pan D. Zhang C.-M. Kirillov S. Hou Y.-M. Cooperman B. S. (2008). Perturbation of the tRNA tertiary core differentially affects specific steps of the elongation cycle. J. Biol. Chem. 283, 18431–18440. 10.1074/jbc.M801560200 18448426 PMC2440604

[B242] Pavlov M. Y. Watts R. E. Tan Z. Cornish V. W. Ehrenberg M. Forster A. C. (2009). Slow peptide bond formation by proline and other N-alkylamino acids in translation. Proc. Natl. Acad. Sci. 106, 50–54. 10.1073/pnas.0809211106 19104062 PMC2629218

[B243] Pedersen S. Bloch P. L. Reeh S. Neidhardt F. C. (1978). Patterns of protein synthesis in *E. coli*: a catalog of the amount of 140 individual proteins at different growth rates. Cell 14, 179–190. 10.1016/0092-8674(78)90312-4 352533

[B244] Peil L. Starosta A. L. Lassak J. Atkinson G. C. Virumäe K. Spitzer M. (2013). Distinct XPPX sequence motifs induce ribosome stalling, which is rescued by the translation elongation factor EF-P. Proc. Natl. Acad. Sci. 110, 15265–15270. 10.1073/pnas.1310642110 24003132 PMC3780873

[B245] Petraitytė G. Preikšaitienė E. Mikštienė V. (2021). Genome editing in medicine: tools and challenges. Acta Medica Litu. 28, 205–219. 10.15388/Amed.2021.28.2.8 PMC9133615 35637939

[B246] Phelps S. S. Jerinic O. Joseph S. (2002). Universally conserved interactions between the ribosome and the anticodon stem-loop of A site tRNA important for translocation. Mol. Cell 10, 799–807. 10.1016/S1097-2765(02)00686-X 12419224

[B247] Phelps S. S. Joseph S. (2005). Non-bridging phosphate oxygen atoms within the tRNA anticodon stem-loop are essential for ribosomal A site binding and translocation. J. Mol. Biol. 349, 288–301. 10.1016/j.jmb.2005.03.079 15890196

[B248] Phizicky E. M. Alfonzo J. D. (2010). Do all modifications benefit all tRNAs? FEBS Lett. 584, 265–271. 10.1016/j.febslet.2009.11.049 19931536 PMC2794898

[B249] Phizicky E. M. Hopper A. K. (2010). tRNA biology charges to the front. Genes Dev. 24, 1832–1860. 10.1101/gad.1956510 20810645 PMC2932967

[B250] Porter J. J. Heil C. S. Lueck J. D. (2021). Therapeutic promise of engineered nonsense suppressor tRNAs. Wiley Interdiscip. Rev. RNA 12, e1641. 10.1002/wrna.1641 33567469 PMC8244042

[B251] Poulis P. Peske F. Rodnina M. V. (2023). The many faces of ribosome translocation along the mRNA: reading frame maintenance, ribosome frameshifting and translational bypassing. Biol. Chem. 404, 755–767. 10.1515/hsz-2023-0142 37077160

[B252] Prabhakar A. Krahn N. Zhang J. Vargas-Rodriguez O. Krupkin M. Fu Z. (2022). Uncovering translation roadblocks during the development of a synthetic tRNA. Nucleic Acids Res. 50, 10201–10211. 10.1093/nar/gkac576 35882385 PMC9561287

[B253] Raftery L. A. Yarus M. (1987). Systematic alterations in the anticodon arm make tRNA^Glu^-Su_raj_ a more efficient suppressor. EMBO J. 6, 1499–1506. 10.1002/j.1460-2075.1987.tb02392.x 3301329 PMC553957

[B254] RajBhandary U. L. (1994). Initiator transfer RNAs. J. Bacteriol. 176, 547–552. 10.1128/jb.176.3.547-552.1994 7507918 PMC205089

[B255] Rasmussen L. C. Laursen B. S. Mortensen K. K. Sperling-Petersen H. U. (2009). “Initiator tRNAs in bacteria and eukaryotes,” in Encyclopedia of life Sciences (John Wiley and Sons, Ltd). 10.1002/9780470015902.a0000543.pub2

[B256] Reich H. J. Hondal R. J. (2016). Why nature chose selenium. ACS Chem. Biol. 11, 821–841. 10.1021/acschembio.6b00031 26949981

[B257] Riddle D. L. Carbon J. (1973). Frameshift suppression: a nucleotide addition in the anticodon of a glycine transfer RNA. Nat. New Biol. 242, 230–234. 10.1038/newbio242230a0 4573868

[B258] Riddle D. L. Roth J. R. (1970). Suppressors of frameshift mutations in *Salmonella typhimurium* . J. Mol. Biol. 54, 131–144. 10.1016/0022-2836(70)90451-1 4321728

[B259] Riyasaty S. Atkins J. F. (1968). External suppression of a frameshift mutant in *Salmonella* . J. Mol. Biol. 34, 541–557. 10.1016/0022-2836(68)90179-4 4938557

[B260] Rodnina M. V. (2018). Translation in prokaryotes. Cold Spring Harb. Perspect. Biol. 10, a032664. 10.1101/cshperspect.a032664 29661790 PMC6120702

[B261] Rodnina M. V. (2023). Decoding and recoding of mRNA sequences by the ribosome. Annu. Rev. Biophys. 52, 161–182. 10.1146/annurev-biophys-101922-072452 37159300

[B262] Rodnina M. V. Fischer N. Maracci C. Stark H. (2017). Ribosome dynamics during decoding. Philos. Trans. R. Soc. B Biol. Sci. 372, 20160182. 10.1098/rstb.2016.0182 PMC5311926 28138068

[B263] Rodnina M. V. Peske F. Peng B.-Z. Belardinelli R. Wintermeyer W. (2020). Converting GTP hydrolysis into motion: versatile translational elongation factor g. Biol. Chem. 401, 131–142. 10.1515/hsz-2019-0313 31600135

[B264] Rodnina M. V. Savelsbergh A. Katunin V. I. Wintermeyer W. (1997). Hydrolysis of GTP by elongation factor g drives tRNA movement on the ribosome. Nature 385, 37–41. 10.1038/385037a0 8985244

[B265] Rogalski M. Karcher D. Bock R. (2008). Superwobbling facilitates translation with reduced tRNA sets. Nat. Struct. Mol. Biol. 15, 192–198. 10.1038/nsmb.1370 18193063

[B266] Rogers J. M. Kwon S. Dawson S. J. Mandal P. K. Suga H. Huc I. (2018). Ribosomal synthesis and folding of peptide-helical aromatic foldamer hybrids. Nat. Chem. 10, 405–412. 10.1038/s41557-018-0007-x 29556052

[B267] Romby P. Carbon P. Westhof E. Ehresmann C. Ebel J.-P. Ehresmann B. (1987). Importance of conserved residues for the conformation of the T-loop in tRNAs. J. Biomol. Struct. Dyn. 5, 669–687. 10.1080/07391102.1987.10506419 3078237

[B268] Romero G. Chau V. Biltonen R. L. (1985). Kinetics and thermodynamics of the interaction of elongation factor tu with elongation factor ts, guanine nucleotides, and aminoacyl-tRNA. J. Biol. Chem. 260, 6167–6174. 10.1016/S0021-9258(18)88952-9 3846595

[B269] Roy B. Liu Q. Shoji S. Fredrick K. (2017). IF2 and unique features of initiator tRNA^fMet^ help establish the translational reading frame. RNA Biol. 15, 604–613. 10.1080/15476286.2017.1379636 28914580 PMC6103701

[B270] Rozov A. Demeshkina N. Westhof E. Yusupov M. Yusupova G. (2016). New structural insights into translational miscoding. Trends biochem. Sci. 41, 798–814. 10.1016/j.tibs.2016.06.001 27372401

[B271] Rudinger J. Hillenbrandt R. Sprinzl M. Giegé R. (1996). Antideterminants present in minihelix(Sec) hinder its recognition by prokaryotic elongation factor tu. EMBO J. 15, 650–657. 10.1002/j.1460-2075.1996.tb00397.x 8599948 PMC449983

[B272] Saks M. E. Conery J. S. (2007). Anticodon-dependent conservation of bacterial tRNA gene sequences. RNA 13, 651–660. 10.1261/rna.345907 17379816 PMC1852809

[B273] Sanbonmatsu K. Y. Joseph S. Tung C.-S. (2005). Simulating movement of tRNA into the ribosome during decoding. Proc. Natl. Acad. Sci. 102, 15854–15859. 10.1073/pnas.0503456102 16249344 PMC1266076

[B274] Sanderson L. E. Uhlenbeck O. C. (2007a). Exploring the specificity of bacterial elongation factor tu for different tRNAs. Biochemistry 46, 6194–6200. 10.1021/bi602548v 17489561

[B275] Sanderson L. E. Uhlenbeck O. C. (2007b). The 51–63 base pair of tRNA confers specificity for binding by EF-Tu. RNA 13, 835–840. 10.1261/rna.485307 17449728 PMC1869040

[B276] Santoro S. W. Anderson J. C. Lakshman V. Schultz P. G. (2003). An archaebacteria‐derived glutamyl‐tRNA synthetase and tRNA pair for unnatural amino acid mutagenesis of proteins in *Escherichia coli* . Nucleic Acids Res. 31, 6700–6709. 10.1093/nar/gkg903 14627803 PMC290271

[B277] Schmeing T. M. Voorhees R. M. Kelley A. C. Gao Y.-G. Murphy F. V. Weir J. R. (2009). The crystal structure of the ribosome bound to EF-Tu and aminoacyl-tRNA. Science 326, 688–694. 10.1126/science.1179700 19833920 PMC3763470

[B278] Schmeing T. M. Voorhees R. M. Kelley A. C. Ramakrishnan V. (2011). How mutations in tRNA distant from the anticodon affect the fidelity of decoding. Nat. Struct. Mol. Biol. 18, 432–436. 10.1038/nsmb.2003 21378964 PMC3072312

[B279] Schmied W. H. Elsässer S. J. Uttamapinant C. Chin J. W. (2014). Efficient multisite unnatural amino acid incorporation in mammalian cells via optimized pyrrolysyl tRNA synthetase/tRNA expression and engineered eRF1. J. Am. Chem. Soc. 136, 15577–15583. 10.1021/ja5069728 25350841 PMC4333590

[B280] Schrader J. M. Chapman S. J. Uhlenbeck O. C. (2009). Understanding the sequence specificity of tRNA binding to elongation factor Tu using tRNA mutagenesis. J. Mol. Biol. 386, 1255–1264. 10.1016/j.jmb.2009.01.021 19452597 PMC2779119

[B281] Schrader J. M. Chapman S. J. Uhlenbeck O. C. (2011). Tuning the affinity of aminoacyl-tRNA to elongation factor tu for optimal decoding. Proc. Natl. Acad. Sci. 108, 5215–5220. 10.1073/pnas.1102128108 21402928 PMC3069205

[B282] Schrader J. M. Uhlenbeck O. C. (2011). Is the sequence-specific binding of aminoacyl-tRNAs by EF-Tu universal among bacteria? Nucleic Acids Res. 39, 9746–9758. 10.1093/nar/gkr641 21893586 PMC3239215

[B283] Schuntermann D. B. Fischer J. T. Bile J. Gaier S. A. Shelley B. A. Awawdeh A. (2023). Mistranslation of the genetic code by a new family of bacterial transfer RNAs. J. Biol. Chem. 299, 104852. 10.1016/j.jbc.2023.104852 37224963 PMC10404621

[B284] Schwartzbach C. J. Spremulli L. L. (1991). Interaction of animal mitochondrial EF-Tu.EF-Ts with aminoacyl-tRNA, guanine nucleotides, and ribosomes. J. Biol. Chem. 266, 16324–16330. 10.1016/S0021-9258(18)55300-X 1885567

[B285] Seong B. L. RajBhandary U. L. (1987). *Escherichia coli* formylmethionine tRNA: mutations in GGGCCC sequence conserved in anticodon stem of initiator tRNAs affect initiation of protein synthesis and conformation of anticodon loop. Proc. Natl. Acad. Sci. 84, 334–338. 10.1073/pnas.84.2.334 3540960 PMC304201

[B286] Serfling R. Lorenz C. Etzel M. Schicht G. Böttke T. Mörl M. (2018). Designer tRNAs for efficient incorporation of non-canonical amino acids by the pyrrolysine system in mammalian cells. Nucleic Acids Res. 46, 1–10. 10.1093/nar/gkx1156 29177436 PMC5758916

[B287] Shafer A. M. Kálai T. Bin Liu S. Q. Hideg K. Voss J. C. (2004). Site-specific insertion of spin-labeled l-amino acids in *Xenopus* oocytes. Biochemistry 43, 8470–8482. 10.1021/bi035542i 15222758

[B288] Shah R. A. Varada R. Sah S. Shetty S. Lahry K. Singh S. (2019). Rapid formylation of the cellular initiator tRNA population makes a crucial contribution to its exclusive participation at the step of initiation. Nucleic Acids Res. 47, 1908–1919. 10.1093/nar/gky1310 30608556 PMC6393288

[B289] Shao S. Murray J. Brown A. Taunton J. Ramakrishnan V. Hegde R. S. (2016). Decoding mammalian ribosome-mRNA states by translational GTPase complexes. Cell 167, 1229–1240. 10.1016/j.cell.2016.10.046 27863242 PMC5119991

[B290] Shepotinovskaya I. Uhlenbeck O. C. (2013). tRNA residues evolved to promote translational accuracy. RNA 19, 510–516. 10.1261/rna.036038.112 23440350 PMC3677261

[B291] Shin B.-S. Katoh T. Gutierrez E. Kim J.-R. Suga H. Dever T. E. (2017). Amino acid substrates impose polyamine, eIF5A, or hypusine requirement for peptide synthesis. Nucleic Acids Res. 45, 8392–8402. 10.1093/nar/gkx532 28637321 PMC5737446

[B292] Sigal M. Matsumoto S. Beattie A. Katoh T. Suga H. (2024). Engineering tRNAs for the ribosomal translation of non-proteinogenic monomers. Chem. Rev. 124, 6444–6500. 10.1021/acs.chemrev.3c00894 38688034 PMC11122139

[B293] Smith T. J. Giles R. N. Koutmou K. S. (2024). Anticodon stem-loop tRNA modifications influence codon decoding and frame maintenance during translation. Semin. Cell Dev. Biol. 154, 105–113. 10.1016/j.semcdb.2023.06.003 37385829 PMC11849751

[B294] Song H. Zhang J. Liu B. Xu J. Cai B. Yang H. (2022). Biological roles of RNA m5C modification and its implications in Cancer immunotherapy. Biomark. Res. 10, 15. 10.1186/s40364-022-00362-8 35365216 PMC8973801

[B295] Soye B. J. D. Patel J. R. Isaacs F. J. Jewett M. C. (2015). Repurposing the translation apparatus for synthetic biology. Curr. Opin. Chem. Biol. 28, 83–90. 10.1016/j.cbpa.2015.06.008 26186264 PMC5113029

[B296] Starosta A. L. Lassak J. Peil L. Atkinson G. C. Virumäe K. Tenson T. (2014). Translational stalling at polyproline stretches is modulated by the sequence context upstream of the stall site. Nucleic Acids Res. 42, 10711–10719. 10.1093/nar/gku768 25143529 PMC4176338

[B297] Stenson P. D. Mort M. Ball E. V. Evans K. Hayden M. Heywood S. (2017). The human gene mutation database: towards a comprehensive repository of inherited mutation data for medical research, genetic diagnosis and next-generation sequencing studies. Hum. Genet. 136, 665–677. 10.1007/s00439-017-1779-6 28349240 PMC5429360

[B298] Strobel S. A. Cech T. R. Usman N. Beigelman L. (1994). The 2,6-diaminopurine riboside.5-methylisocytidine wobble base pair: an isoenergetic substitution for the study of G.U pairs in RNA. Biochemistry 33, 13824–13835. 10.1021/bi00250a037 7524665

[B299] Sun F.-J. Caetano-Anollés G. (2009). The evolutionary significance of the long variable arm in transfer RNA. Complexity 14, 26–39. 10.1002/cplx.20255

[B300] Süssmuth R. D. Mainz A. (2017). Nonribosomal peptide synthesis—principles and prospects. Angew. Chem. Int. Ed. 56, 3770–3821. 10.1002/anie.201609079 28323366

[B301] Suzuki T. (2021). The expanding world of tRNA modifications and their disease relevance. Nat. Rev. Mol. Cell Biol. 22, 375–392. 10.1038/s41580-021-00342-0 33658722

[B302] Suzuki T. Yashiro Y. Kikuchi I. Ishigami Y. Saito H. Matsuzawa I. (2020). Complete chemical structures of human mitochondrial tRNAs. Nat. Commun. 11, 4269. 10.1038/s41467-020-18068-6 32859890 PMC7455718

[B303] Tajima K. Katoh T. Suga H. (2022). Drop-off-reinitiation triggered by EF-G-driven mistranslocation and its alleviation by EF-P. Nucleic Acids Res. 50, 2736–2753. 10.1093/nar/gkac068 35188576 PMC8934632

[B304] Taki M. Matsushita J. Sisido M. (2006). Expanding the genetic code in a mammalian cell line by the introduction of four-base codon/anticodon pairs. ChemBioChem 7, 425–428. 10.1002/cbic.200500360 16440374

[B305] Tamaki S. Tomita M. Suzuki H. Kanai A. (2018). Systematic analysis of the binding surfaces between tRNAs and their respective aminoacyl tRNA synthetase based on structural and evolutionary data. Front. Genet. 8, 227. 10.3389/fgene.2017.00227 29358943 PMC5766645

[B306] Terasaka N. Hayashi G. Katoh T. Suga H. (2014). An orthogonal ribosome-tRNA pair via engineering of the peptidyl transferase center. Nat. Chem. Biol. 10, 555–557. 10.1038/nchembio.1549 24907900

[B307] Terns M. P. (2018). CRISPR-based technologies: impact of RNA-targeting systems. Mol. Cell 72, 404–412. 10.1016/j.molcel.2018.09.018 30388409 PMC6239212

[B308] Tharp J. M. Ad O. Amikura K. Ward F. R. Garcia E. M. Cate J. H. D. (2020a). Initiation of protein synthesis with non-canonical amino acids *in vivo* . Angew. Chem. Int. Ed. 59, 3122–3126. 10.1002/anie.201914671 31828898

[B309] Tharp J. M. Ehnbom A. Liu W. R. (2017). tRNA^Pyl^: structure, function, and applications. RNA Biol. 15, 441–452. 10.1080/15476286.2017.1356561 28837402 PMC6103707

[B310] Tharp J. M. Krahn N. Varshney U. Söll D. (2020b). Hijacking translation initiation for synthetic biology. ChemBioChem 21, 1387–1396. 10.1002/cbic.202000017 32023356 PMC7237318

[B311] Tharp J. M. Vargas-Rodriguez O. Schepartz A. Söll D. (2021a). Genetic encoding of three distinct noncanonical amino acids using reprogrammed initiator and nonsense codons. ACS Chem. Biol. 16, 766–774. 10.1021/acschembio.1c00120 33723984 PMC8336083

[B312] Tharp J. M. Walker J. A. Söll D. Schepartz A. (2021b). Initiating protein synthesis with noncanonical monomers *in vitro* and *in vivo* . Methods Enzymol. 656, 495–519. 10.1016/bs.mie.2021.05.002 34325796 PMC8428779

[B313] Thyer R. Robotham S. A. Brodbelt J. S. Ellington A. D. (2015). Evolving tRNA^Sec^ for efficient canonical incorporation of selenocysteine. J. Am. Chem. Soc. 137, 46–49. 10.1021/ja510695g 25521771 PMC4432777

[B314] Tsiamantas C. Kwon S. Rogers J. M. Douat C. Huc I. Suga H. (2020). Ribosomal incorporation of aromatic oligoamides as peptide sidechain appendages. Angew. Chem. Int. Ed. 59, 4860–4864. 10.1002/anie.201914654 PMC7496375 31894626

[B315] Ude S. Lassak J. Starosta A. L. Kraxenberger T. Wilson D. N. Jung K. (2013). Translation elongation factor EF-P alleviates ribosome stalling at polyproline stretches. Science 339, 82–85. 10.1126/science.1228985 23239623

[B316] Uhlenbeck O. C. Schrader J. M. (2018). Evolutionary tuning impacts the design of bacterial tRNAs for the incorporation of unnatural amino acids by ribosomes. Curr. Opin. Chem. Biol. 46, 138–145. 10.1016/j.cbpa.2018.07.016 30059836 PMC6601615

[B317] Väre V. Y. P. Eruysal E. R. Narendran A. Sarachan K. L. Agris P. F. (2017). Chemical and conformational diversity of modified nucleosides affects tRNA structure and function. Biomolecules 7, 29. 10.3390/biom7010029 28300792 PMC5372741

[B318] Varshavsky A. (2011). The N-end rule pathway and regulation by proteolysis. Protein Sci. 20, 1298–1345. 10.1002/pro.666 21633985 PMC3189519

[B319] Varshney U. Lee C. P. RajBhandary U. L. (1993). From elongator tRNA to initiator tRNA. Proc. Natl. Acad. Sci. 90, 2305–2309. 10.1073/pnas.90.6.2305 8460138 PMC46075

[B320] Walker S. E. Fredrick K. (2006). Recognition and positioning of mRNA in the ribosome by tRNAs with expanded anticodons. J. Mol. Biol. 360, 599–609. 10.1016/j.jmb.2006.05.006 16730356 PMC2602952

[B321] Walsh C. T. O’Brien R. V. Khosla C. (2013). Nonproteinogenic amino acid building blocks for nonribosomal peptide and hybrid polyketide scaffolds. Angew. Chem. Int. Ed. Engl. 52, 7098–7124. 10.1002/anie.201208344 23729217 PMC4634941

[B322] Wan W. Huang Y. Wang Z. Russell W. K. Pai P.-J. Russell D. H. (2010). A facile system for genetic incorporation of two different noncanonical amino acids into one protein in *Escherichia coli* . Angew. Chem. Int. Ed. 49, 3211–3214. 10.1002/anie.201000465 20340150

[B323] Wan W. Tharp J. M. Liu W. R. (2014). Pyrrolysyl-tRNA synthetase: an ordinary enzyme but an outstanding genetic code expansion tool. Biochim. Biophys. Acta 1844, 1059–1070. 10.1016/j.bbapap.2014.03.002 24631543 PMC4016821

[B324] Wang J. Caban K. Gonzalez R. L. (2015). Ribosomal initiation complex-driven changes in the stability and dynamics of initiation factor 2 regulate the fidelity of translation initiation. J. Mol. Biol. 427, 1819–1834. 10.1016/j.jmb.2014.12.025 25596426 PMC4411560

[B325] Wang J. Kwiatkowski M. Forster A. C. (2016a). Kinetics of tRNA^Pyl^-mediated amber suppression in *Escherichia coli* translation reveals unexpected limiting steps and competing reactions. Biotechnol. Bioeng. 113, 1552–1559. 10.1002/bit.25917 26705134

[B326] Wang J. Shin B.-S. Alvarado C. Kim J.-R. Bohlen J. Dever T. E. (2022a). Rapid 40S scanning and its regulation by mRNA structure during eukaryotic translation initiation. Cell 185, 4474–4487.e17. 10.1016/j.cell.2022.10.005 36334590 PMC9691599

[B327] Wang K. Sachdeva A. Cox D. J. Wilf N. M. Lang K. Wallace S. (2014). Optimized orthogonal translation of unnatural amino acids enables spontaneous protein double-labelling and FRET. Nat. Chem. 6, 393–403. 10.1038/nchem.1919 24755590 PMC4430801

[B328] Wang L. Lin S. (2023). Emerging functions of tRNA modifications in mRNA translation and diseases. J. Genet. Genomics 50, 223–232. 10.1016/j.jgg.2022.10.002 36309201

[B329] Wang L. Schultz P. G. (2001). A general approach for the generation of orthogonal tRNAs. Chem. Biol. 8, 883–890. 10.1016/s1074-5521(01)00063-1 11564556

[B330] Wang L. Wang N. Zhang W. Cheng X. Yan Z. Shao G. (2022b). Therapeutic peptides: current applications and future directions. Signal Transduct. Target. Ther. 7, 48–27. 10.1038/s41392-022-00904-4 35165272 PMC8844085

[B331] Wang N. Shang X. Cerny R. Niu W. Guo J. (2016b). Systematic evolution and study of UAGN decoding tRNAs in a genomically recoded bacteria. Sci. Rep. 6, 21898. 10.1038/srep21898 26906548 PMC4764823

[B332] Wang Y. Jiang Y. Meyering-Voss M. Sprinzl M. Sigler P. B. (1997). Crystal structure of the EF-Tu˙EF-Ts complex from *Thermus thermophilus* . Nat. Struct. Biol. 4, 650–656. 10.1038/nsb0897-650 9253415

[B333] Wang Y. Xue P. Cao M. Yu T. Lane S. T. Zhao H. (2021). Directed evolution: methodologies and applications. Chem. Rev. 121, 12384–12444. 10.1021/acs.chemrev.1c00260 34297541

[B334] Ward C. Beharry A. Tennakoon R. Rozik P. Wilhelm S. D. P. Heinemann I. U. (2024). Mechanisms and delivery of tRNA therapeutics. Chem. Rev. 10.1021/acs.chemrev.4c00142 PMC11212642 38801719

[B335] Weissbach H. Miller D. L. Hachmann J. (1970). Studies on the role of factor ts in polypeptide synthesis. Arch. Biochem. Biophys. 137, 262–269. 10.1016/0003-9861(70)90433-9 4907806

[B336] Westhof E. Thornlow B. Chan P. P. Lowe T. M. (2022). Eukaryotic tRNA sequences present conserved and amino acid-specific structural signatures. Nucleic Acids Res. 50, 4100–4112. 10.1093/nar/gkac222 35380696 PMC9023262

[B337] Wohlgemuth I. Brenner S. Beringer M. Rodnina M. V. (2008). Modulation of the rate of peptidyl transfer on the ribosome by the nature of substrates. J. Biol. Chem. 283, 32229–32235. 10.1074/jbc.M805316200 18809677

[B338] Woolstenhulme C. J. Guydosh N. R. Green R. Buskirk A. R. (2015). High-precision analysis of translational pausing by ribosome profiling in bacteria lacking EFP. Cell Rep. 11, 13–21. 10.1016/j.celrep.2015.03.014 25843707 PMC4835038

[B339] Wright D. E. O’Donoghue P. (2024). Biosynthesis, engineering, and delivery of selenoproteins. Int. J. Mol. Sci. 25, 223. 10.3390/ijms25010223 PMC10778597 38203392

[B340] Wu X.-Q. RajBhandary U. L. (1997). Effect of the amino acid attached to *Escherichia coli* Initiator tRNA on its affinity for the initiation factor IF2 and on the IF2 dependence of its binding to the ribosome. J. Biol. Chem. 272, 1891–1895. 10.1074/jbc.272.3.1891 8999877

[B341] Xie J. Schultz P. G. (2006). A chemical toolkit for proteins--an expanded genetic code. Nat. Rev. Mol. Cell Biol. 7, 775–782. 10.1038/nrm2005 16926858

[B342] Xu B. Liu L. Song G. (2022). Functions and regulation of translation elongation factors. Front. Mol. Biosci. 8, 816398. 10.3389/fmolb.2021.816398 35127825 PMC8807479

[B343] Yamagishi Y. Shoji I. Miyagawa S. Kawakami T. Katoh T. Goto Y. (2011). Natural product-like macrocyclic N-methyl-peptide inhibitors against a ubiquitin ligase uncovered from a ribosome-expressed *de novo* library. Chem. Biol. 18, 1562–1570. 10.1016/j.chembiol.2011.09.013 22195558

[B344] Yared M.-J. Marcelot A. Barraud P. (2024). Beyond the anticodon: tRNA core modifications and their impact on structure, translation and stress adaptation. Genes 15, 374. 10.3390/genes15030374 38540433 PMC10969862

[B345] Yarus M. (1982). Translational efficiency of transfer RNA’s: uses of an extended anticodon. Science 218, 646–652. 10.1126/science.6753149 6753149

[B346] Yarus M. Cline S. Raftery L. Wier P. Bradley D. (1986). The translational efficiency of tRNA is a property of the anticodon arm. J. Biol. Chem. 261, 10496–10505. 10.1016/S0021-9258(18)67412-5 3525546

[B347] Yikilmaz E. Chapman S. J. Schrader J. M. Uhlenbeck O. C. (2014). The interface between *Escherichia coli* elongation factor tu and aminoacyl-tRNA. Biochemistry 53, 5710–5720. 10.1021/bi500533x 25094027 PMC4159200

[B348] Yoshizawa S. Böck A. (2009). The many levels of control on bacterial selenoprotein synthesis. Biochim. Biophys. Acta 1790, 1404–1414. 10.1016/j.bbagen.2009.03.010 19328835

[B349] Young D. D. Schultz P. G. (2018). Playing with the molecules of life. ACS Chem. Biol. 13, 854–870. 10.1021/acschembio.7b00974 29345901 PMC6061972

[B350] Young T. S. Ahmad I. Yin J. A. Schultz P. G. (2010). An enhanced system for unnatural amino acid mutagenesis in *E. coli* . J. Mol. Biol. 395, 361–374. 10.1016/j.jmb.2009.10.030 19852970

[B351] Yourno J. Kohno T. (1972). Externally suppressible proline quadruplet CCCU. Science 175, 650–652. 10.1126/science.175.4022.650 5009765

[B352] Yusupov M. M. Yusupova G.Zh. Baucom A. Lieberman K. Earnest T. N. Cate J. H. D. (2001). Crystal structure of the ribosome at 5.5 Å resolution. Science 292, 883–896. 10.1126/science.1060089 11283358

[B353] Zhang D. Zhu L. Wang F. Li P. Wang Y. Gao Y. (2023). Molecular mechanisms of eukaryotic translation fidelity and their associations with diseases. Int. J. Biol. Macromol. 242, 124680. 10.1016/j.ijbiomac.2023.124680 37141965

[B354] Zhang J. Ferré-D’Amaré A. R. (2016). The tRNA elbow in structure, recognition and evolution. Life Basel Switz. 6, 3. 10.3390/life6010003 PMC4810234 26771646

[B355] Zhang W. Foo M. Eren A. M. Pan T. (2022). tRNA modification dynamics from individual organisms to metaepitranscriptomics of microbiomes. Mol. Cell 82, 891–906. 10.1016/j.molcel.2021.12.007 35032425 PMC8897278

[B356] Zhao H. Ding W. Zang J. Yang Y. Liu C. Hu L. (2021). Directed-evolution of translation system for efficient unnatural amino acids incorporation and generalizable synthetic auxotroph construction. Nat. Commun. 12, 7039. 10.1038/s41467-021-27399-x 34857769 PMC8639764

[B357] Zheng Y. Gilgenast M. J. Hauc S. C. Chatterjee A. (2018). Capturing post-translational modification-triggered protein-protein interactions using dual noncanonical amino acid mutagenesis. ACS Chem. Biol. 13, 1137–1141. 10.1021/acschembio.8b00021 29544052 PMC6446081

[B358] Zhou D. Lee J. Frankenberger C. Geslain R. Rosner M. Pan T. (2012). Anti-tumor effects of an engineered “killer” transfer RNA. Biochem. Biophys. Res. Commun. 427, 148–153. 10.1016/j.bbrc.2012.09.028 22989754

[B359] Zhou J. Lancaster L. Donohue J. P. Noller H. F. (2014). How the ribosome hands the A-site tRNA to the P Site during EF-G-catalyzed translocation. Science 345, 1188–1191. 10.1126/science.1255030 25190797 PMC4242719

